# Marker Metabolite‐Based Multi‐Omics Analysis Identifies New Loci Controlling Thousand Seed Weight in *Brassica Napus*

**DOI:** 10.1002/advs.202512509

**Published:** 2025-09-19

**Authors:** Long Li, Zhitao Tian, Xiaowei Wu, Zengdong Tan, Xu Han, Yuyan Xiang, Jie Chen, Hu Zhao, Wei Chen, Liang Guo, Xuan Yao

**Affiliations:** ^1^ National Key Laboratory of Crop Genetic Improvement Hubei Hongshan Laboratory Huazhong Agricultural University Wuhan 430070 China; ^2^ Department of Microbiology Harvard Medical School Boston MA 02115 USA; ^3^ Yazhouwan National Laboratory Sanya 572025 China

**Keywords:** brassica napus, CUT&Tag, dual‐luciferase assay, EMSA, marker metabolite, metabolome, mGWAS, mTWAS, thousand seed weight

## Abstract

The thousand seed weight (TSW) of *Brassica napus* (*B. napus*) is a crucial agronomic trait, and metabolites serve as a bridge between genotype and phenotype. Leveraging previously released metabolome data from a 388 *B. napus* population, 137 marker metabolites significantly correlated with TSW are identified, and 21 markers significantly correlate with both TSW and seed oil content. To delve deeper into the relationships between selected marker metabolites and phenotypes, a comprehensive multi‐omics analysis is conducted, which unveils 734 metabolite‐QTLs, 5,225 expression‐QTLs, and 9,077 transcriptome‐wide significantly associated genes. Employing weighted correlation network analysis, a triple‐link network is constructed involving 133 metabolites, 731 QTLs, and 3,321 genes. The multi‐omics analysis highlights the impact of the transcriptional factor TGACG motif‐binding factor 6 (BnaA03.TGA6) on TSW. Functional validation experiments with *tga6* CRISPR/Cas9 mutants demonstrate larger seeds and altered associated metabolites levels compared to the wild type. RNA Sequencing, dual‐luciferase assay, Cleavage Under Targets and Tagmentation, and Electrophoretic Mobility Shift Assay results confirm that BnaA03.TGA6 activates the expression of *E3 ubiquitin‐protein ligase DA2*. In conclusion, this study is the first marker metabolite‐based multi‐omics analysis in unraveling the genetic basis of TSW in crop and identifying *BnaA03.TGA6* as a novel gene negatively influencing TSW in *B. napus*.

## Graphical abstract

Marker metabolite‐based multi‐omics (genome, transcriptome, and metabolome) analysis is conducted firstly to unravel the genetic basis of thousand seed weight (TSW) in Brassica napus. The released metabolite‐QTL‐gene network represents a valuable genetic resource, and the newly validated BnaTGA6 is a promising target for the improvement of TSW in Brassica napus.

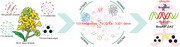

## 1 Introduction

More than 200000 types of metabolites are estimated to exist in plants.^[^ ^1^, ^2^ ^]^ Typically, variations in plant growth or responses to stresses are accompanied by the decrease or increase in specific metabolites. These compounds serve as valuable markers or indicators for distinct biological processes or physical traits.^[^ ^3^, ^4^, ^5^, ^6^, ^7^ ^]^ Plant metabolites play a pivotal role in environmental defense and adaptation, essential for plant growth and development.^[^ ^8^, ^9^ ^]^ Numerous studies have linked certain metabolites strongly to physical traits,^[^ ^6^, ^7^ ^]^ such as their role in regulating maize lignin content to increase plant height and biomass,^[^ ^10^ ^]^ the discovery of amino acid metabolites as markers for improving soybean seed oil content (SOC) or seed protein content,^[^ ^11^ ^]^ and the identification of flavonoid metabolites as markers for SOC in *Brassica napus* (*B. napus*).^[^ ^12^ ^]^ Given that secondary metabolites are more diverse compared to primary metabolites,^[^ ^13^, ^14^ ^]^ their utilization, along with genome and transcriptome data, has led to an increasing number of studies showcasing how metabolomics advances the exploration of crop metabolic sciences.^[^ ^15^, ^16^, ^17^, ^18^ ^]^

QTL mapping is widely used in the agronomic loci and thousand‐seed weight (TSW) related gene discovery.^[^ ^19^, ^20^ ^]^ Such as *Auxin‐response factor 18* (*BnaA09.ARF18*) is mapped at a major QTL on A09,^[^ ^21^ ^]^ P450 monooxygenase gene *BnaA9.CYP78A9* also on A09 was mapped by a transposable element region controlling seed weight and silique length in *B. napus*.^[^ ^22^ ^]^ And *shaggy‐like protein kinase 41* (*SK41*) is found at QTL *qTGW3*, controlling seed size and weight in rice.^[^ ^23^ ^]^ With the development of *de novo* sequencing, genome‐wide association studies (GWAS) have been identifying more novel loci.^[^ ^24^, ^25^ ^]^ For example, *GmWRI14‐like* genes were identified to control soybean seed weight,^[^ ^26^ ^]^ and flavin‐binding *monooxygenase family* proteins in peanut are associated with seed weight.^[^ ^27^ ^]^ Metabolome‐based genome‐wide association studies (mGWAS) have successfully unearthed numerous loci linked to pivotal agronomic traits in major crops, such as rice, peach, sesame, and lettuce.^[^ ^16^, ^17^, ^28^, ^29^, ^30^, ^31^, ^32^, ^33^, ^34^ ^]^ In tandem with these identified loci, metabolome‐based transcriptome‐wide association studies (mTWAS) aim to elucidate gene‐metabolite relationships through comprehensive association analysis.^[^ ^12^, ^35^, ^36^, ^37^, ^38^, ^39^ ^]^ The integration of transcriptome and genome data in expression genome‐wide association studies (eGWAS) is designed to pinpoint genomic loci influencing gene transcript levels.^[^ ^12^, ^40^ ^]^ Consequently, a multi‐omics analysis incorporating genome, transcriptome, and metabolome data holds significant promise for advancing the discovery and utilization of loci and genes associated with crop traits.

TSW is one of the three key yield determinants for *B. napus*,^[^ ^41^ ^]^ which is a global secondary oil crop providing ≈13% of edible oil (USDA ERS, 2022). Recent investigations have unveiled several pathways governing seed weight, including the involvement of the *ubiquitin receptor DA2* in the ubiquitin‐proteasome pathway,^[^ ^42^ ^]^ Arabidopsis *G protein gamma subunit 3* (*AGG3*) in G‐protein signaling,^[^ ^43^, ^44^ ^]^ and *mitogen‐activated protein kinase phosphatase1* (*MKP1*), *mitogen‐activated protein kinase 6* (*MKP6*), and *mitogen‐activated protein kinase kinase 5* (*MKK5*) in MAPK signaling.^[^ ^45^, ^46^, ^47^, ^48^, ^49^ ^]^ *DNA METHYLTRANSFERASE1* (*MET1*) and *DECREASE IN DNA METHYLATION1* (*DDM1*) exhibit parent‐of‐origin effects.^[^ ^50^ ^]^ Notably, downregulating the ubiquitin receptor *BnaDA1* has been demonstrated to increase *B. napus* seed size.^[^ ^51^ ^]^

Numerous transcription factors (TFs) influencing seed weight have been reported.^[^ ^52^, ^53^ ^]^ In Arabidopsis, the chromatin status of TRANSPARENT TESTA GLABRA2 (TTG2), regulated by TOPOISOMERASE Iα (TOP1‐α), is instrumental in determining seed size.^[^ ^54^, ^55^ ^]^ Over‐expression of *Leafy Cotyledon 1 (LEC1)* and *WRINKLED 1 (WRI1)* from *Pistacia chinensis* in Arabidopsis has been shown to enhance seed weight.^[^ ^56^ ^]^ *HECT E3 ubiquitin ligase* (*BnaC03.UPL3*) has been demonstrated to directly impact the protein level of *Leafy Cotyledon 2* (*LEC2*), exerting a negative influence on both TSW and SOC.^[^ ^57^ ^]^ In soybeans, the down‐regulation of *BIG SEEDS1* (*BS1*), a plant‐specific TF, significantly increases seed size.^[^ ^58^ ^]^ *DNA binding with one finger* (*Dof*) and *GmWRI14‐like genes* also play roles in influencing seed weight in soybean.^[^ ^26^, ^59^ ^]^ On the other hand, *TGA6*, a transcription factor possessing a basic region domain leucine zipper domain (b‐ZIP) and belonging to the second branch of the Arabidopsis TGA family,^[^ ^60^ ^]^ has been reported to directly bind to the promoter sequence of *PATHOGENESIS‐RELATED1*.^[^ ^61^ ^]^ It participates in activating the salicylic acid response gene alongside *TGA2* and *TGA5*.^[^ ^62^ ^]^ TGA6 inhibits immune response through physical interaction in the nucleus.^[^ ^63^ ^]^ However, its impact on TSW has not been previously reported in plants.

A comprehensive analysis quantified 2172 metabolites in mature seeds of *B. napus* in our previous work.^[^ ^12^ ^]^ This is the first metabolome‐coupled multi‐omics cohort study that reveals 137 marker metabolites significantly associated with TSW by computing correlation coefficients between TSW and the metabolome. Utilizing multi‐omics analysis and weighted correlation network analysis (WGCNA), we construct a ternary relationship network linking metabolites, genes, and QTLs to TSW. Our findings identify 30 seed size‐related candidate genes, including *Auxin response factor 2* and *AP2‐like ethylene‐responsive transcription factor ANT*. Experimental validation confirms the feasibility and effectiveness of our approach. Notably, *BnaA03.TGA6* emerges as a novel transcription factor influencing TSW in *B. napus*.

## 2 Results

### 2.1 Metabolic Profiling Identifies 137 Marker Metabolites of TSW in *B. Napus*

**Figure 1:**
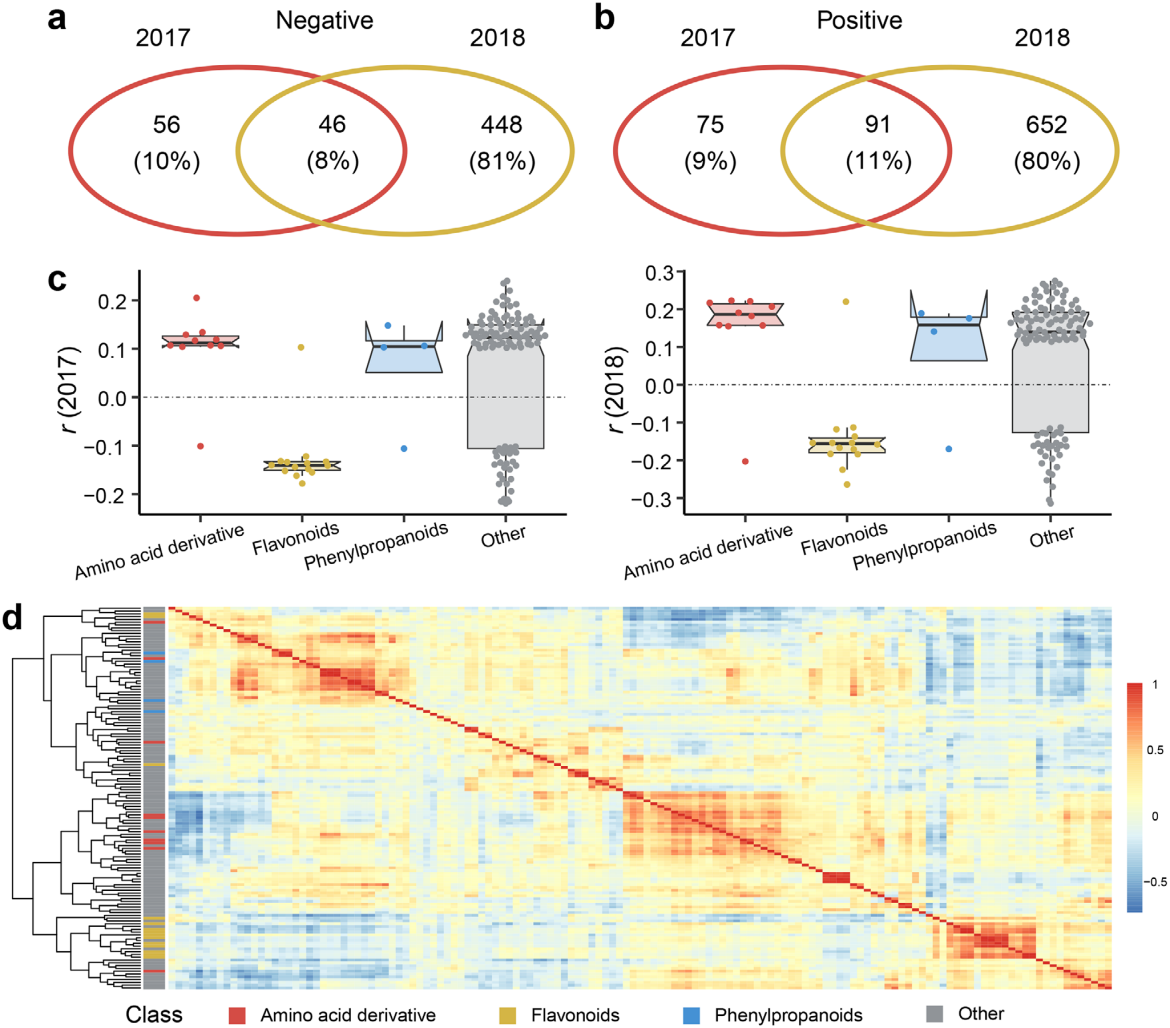
*B. napus* TSW and metabolome data in two years. a) The Venn diagram of metabolites negatively correlated with TSW in two years. b) The Venn diagram of metabolites positively correlated with TSW in two years. c) The correlation index of 137 TSW‐correlated metabolites in two years. d) The heatmap of different classes of 137 TSW‐correlated metabolites in the natural population. The color of each cell represents the correlation levels with each TSW‐correlated metabolite.

In our prior investigation, a comprehensive metabolomics approach quantified a total of 2172 metabolites in mature seeds from 388 *B. napus* inbred lines.^[^ ^12^ ^]^ To explore marker metabolites associated with TSW in *B. napus*, we have conducted a corresponding calculation of the correlation between TSW and metabolites. The correlation indices are computed separately for two consecutive years, revealing 46 consistently negatively correlated TSW marker metabolites (**Figure** **1**a) and 91 consistently positively correlated ones across two years (Figure 1b). Consequently, 137 metabolites across two years (*p* < 0.05) are identified as TSW marker metabolites (Table S1, Supporting Information). These marker metabolites are classified based on NPClassifier,^[^ ^64^ ^]^ including 14 flavonoid metabolites, 10 amino acid derivatives, 4 phenylpropanoid metabolites, and 109 unknown metabolites. The correlation indices among TSW marker metabolites over the two years demonstrate good repeatability (Figure 1c). Furthermore, correlation analysis for the 137 TSW marker metabolites reveals high inter‐class correlation among metabolites within the same class (Figure 1d). The average heritability value of TSW marker metabolites is 0.67, surpassing the average value of all detected metabolites (Figure S1, Supporting Information).

### 2.2 Multi‐Omics Analysis Reveals QTLs Potentially Regulating TSW

**Figure 2:**
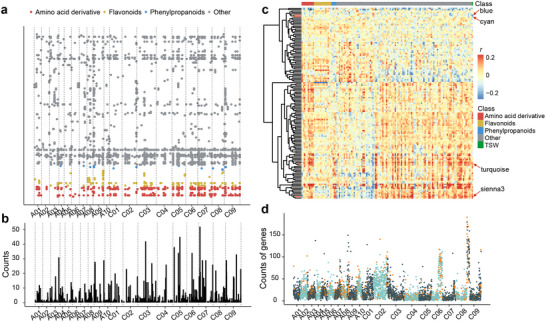
mGWAS and module‐trait association studies of TSW and TSW‐correlated metabolites. a) Chromosomal distribution of all lead variations associated with TSW‐correlated metabolites (the results of 2017 and 2018 are combined). b) hromosomal distribution of all mQTLs associated with TSW‐correlated metabolites (the results of 2017 and 2018 are combined). The interval size is 100 kb. c) Heatmap of correlation among TSW‐correlated metabolites, TSW, and eigenvalues of modules obtained by WGCNA. Each eigenvalue represents the profile of the genes in the correlated module. The vertical axis shows the modules. d) The genome‐wide distribution of eQTLs. The X‐axis represents the physical position of the eQTLs, and the Y‐axis represents the number of genes associated with the eQTL. The orange point indicates that the eQTL colocalizes with the mQTL detected by mGWAS of TSW‐correlated metabolites.

mGWAS analysis has been conducted on the 137 TSW marker metabolites,^[^ ^12^ ^]^ resulting in the detection of 1154 significantly associated variations for 133 TSW marker metabolites (**Figure** **2**a; Table S2, Supporting Information). Notably, 111 significant signals are identified for 13 flavonoids, 27 for 4 phenylpropanoid metabolites, 183 for 9 amino acid derivatives, and 1046 for 107 unknown metabolites (Figure 2a; Table S2, Supporting Information). Utilizing a linkage disequilibrium (LD) threshold of 100 kb, these significantly associated loci are categorized into 734 metabolite‐QTLs (mQTLs). These mQTLs colocalization with more than 30 signals are distributed on chromosomes A03, C03, C05, C06, C07, and C09 (Figure 2b; Table S2, Supporting Information). These results indicate that there are obvious mQTL hotspots of TSW marker metabolites.

In our prior research, transcriptome data from seeds developed 40 days after flowering (DAF) were obtained.^[^ ^37^ ^]^ To enhance our understanding of the relationship between transcripts and TSW marker metabolites, mTWAS has been performed on the 40 DAF transcriptome datasets, revealing 9077 genes significantly associated with TSW marker metabolites (*p* < 1.41 × 10^−05^; Table S3, Supporting Information). WGCNA has been executed using the transcripts from the 40 DAF transcriptome data, clustering them into 139 modules. Correlation coefficients of these modules with both TSW and TSW marker metabolites have been calculated (Figure 2c; Table S4, Supporting Information). Among these modules, 19 exhibit significant correlations with TSW (*p* < 0.01), with module sienna3 displaying the strongest correlation (Table S5, Supporting Information). GO enrichment analysis reveals that module sienna3 is enriched in cell fate determination and seed coat development (Figure S2a, Supporting Information), while other modules, such as blue, cyan, and turquoise are enriched in pathways such as lipid metabolism and seed development (Figure S2b–d, Supporting Information). These findings underscore the utility of gene co‐expression networks in unveiling candidate gene sets correlated to TSW.

To elucidate the connection between genomic variation and gene expression, we have conducted eGWAS on the 9077 genes significantly correlated with TSW marker metabolites. The eGWAS reveals 59121 variations significantly associated with these genes. Employing a linkage disequilibrium (LD) threshold of 100 kb, we have categorized these variations into 5225 expression‐QTLs (eQTLs) (Table S6, Supporting Information). Notably, eQTL hotspot analysis highlights specific genomic regions, particularly on chromosomes A02, A03, A05, A09, C01, C02, C03, C06, C07, and C09, where eQTLs are associated with over 100 genes (Figure 2d). This observation implies the presence of transcription regulatory factors in these eQTL hotspots, potentially manipulating a cluster of genes that directly or indirectly influence TSW marker metabolite content.

### 2.3 A Seed Development‐Related Gene *BnaC09.DDM1* is Significantly Associated with TSW

**Figure 3:**
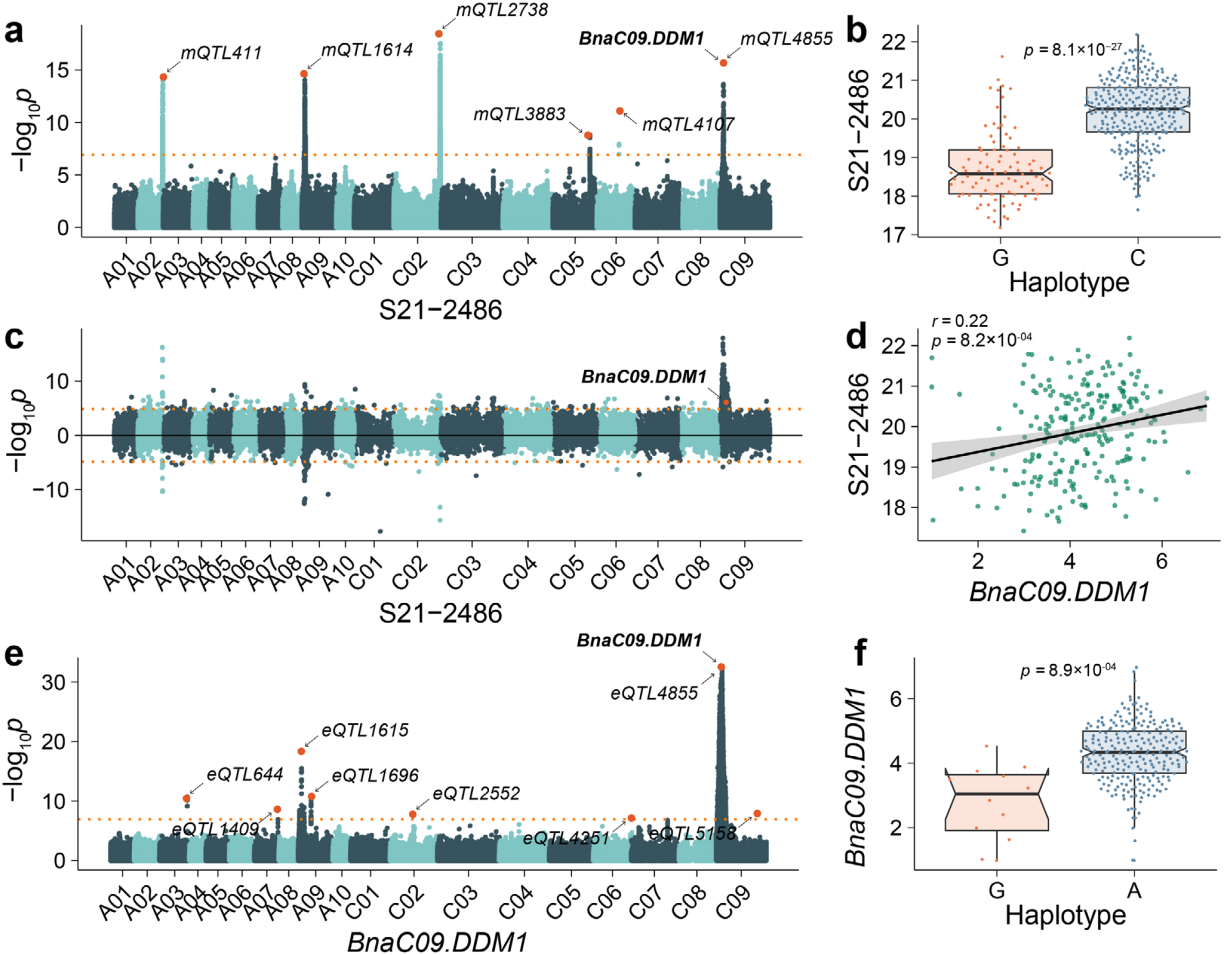
Multi‐omics identifies *BnaC09.DDM1* as TSW candidate gene. a) Manhattan plot of mGWAS for S21‐2486. b) Haplotype analysis of the lead variation in *mQTL4855* for S21–2486 content. c) Manhattan plot of mTWAS for S21–2486. d) Correlation analysis between S21 and 2486 content and *BnaC09.DDM1* expression level. e) Manhattan plot of eGWAS for *BnaC09.DDM1*. f) Haplotype analysis of the lead variation in *eQTL4855* for *BnaC09.DDM1* expression level.

DECREASED DNA METHYLATION 1 (DDM1) plays a pivotal role as an ATP‐dependent chromatin remodeler crucial for maintaining DNA methylation in plants, a process essential for seed development.^[^ ^50^ ^]^ In our study, *BnaC09.DDM1* (*BnaC09g07810D*) is predicted to be significantly associated with TSW‐correlated metabolites. Six mQTLs are identified to be associated with S21–2486 (**Figure** **3**a), and haplotype analysis within *mQTL4855* (Table s7, Supporting Information) reveals that, at the population level, inbred lines with haplotype C exhibit higher S21–2486 content in seeds (*p* = 8.1 × 10^−27^; Figure 3b). Since it's a TSW correlated metabolite QTL hotspots, all variations, position and genomic region information has been summarized in Table S7 (Supporting Information). mTWAS analysis demonstrates a significant association between *BnaC09.DDM1* and S21–2486 content (Figure 3c). Furthermore, the expression levels of *BnaC09.DDM1* show a significant correlation with S21–2486 (r = 0.22, *p* = 8.2 × 10^−04^; Figure 3d). eGWAS results indicate that the expression level of *BnaC09.DDM1* is significantly associated with 1 *cis*‐eQTL and 7 *trans*‐eQTLs (Figure 3e). Haploid analysis reveals that haplotype A represents a high expression level of *BnaC09.DDM1* (*p* = 8.9 × 10^−04^, Figure 3f; Table S6, Supporting Information). Notably, *BnaC09.DDM1* is associated with 14 metabolites whose 18 mQTLs are co‐located on the C09 chromosome, suggesting the identification of a mQTL hotspot with potential influence on TSW (Table S2, Supporting Information). Additionally, mTWAS results highlight the significant association of *BnaC09.DDM1* with three metabolites: wm0034 (*p* = 7.23 × 10^−06^), S21–2486 (*p* = 1.13 × 10^−06^), and S21_L‐3616 (*p* = 3.08 × 10^−06^) (Table S3, Supporting Information). We have annotated wm0034 as 4‐indolecarbaldehyde and provided high‐resolution spectra for the other two unknown metabolites (Figure S3, Supporting Information). These findings collectively position *BnaC09.DDM1* as a promising candidate gene influencing TSW.

### 2.4 Ternary Relationship Networks Boost the Understanding of TSW and SOC

**Figure 4:**
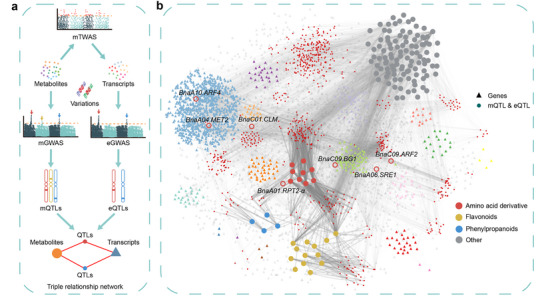
TSW‐correlated metabolites, genes, and QTLs network. a) Flow chart of network building. b) Network built based on the correlation among TSW‐correlated metabolites, genes, and QTLs. Candidate genes, *BnaA10.ARF4*, *BnaA04.MET2*, *BnaC01.CLM*, *BnaA01.RPT2‐α*, *BnaC09.BG1*, *BnaA06.SRE1*, and *BnaC09.ARF2* are shown as circles with a distinct color per co‐expression module. All modules significantly correlated with TSW are shown in this network. The QTLs are detected simultaneously by the significant associated genes (eQTL) and metabolites (mQTL) in this triple relationship network.

To elucidate our network‐building approach, we have outlined a comprehensive flowchart (**Figure** **4**a). The outcomes of mGWAS and transcriptome analysis have been amalgamated into a ternary relationship network (gene‐QTL‐metabolite) (Figure 4b; Table S7, Supporting Information), encompassing 731 QTLs, representing both mQTLs and eQTLs, 3321 genes, and 133 TSW marker metabolites. Based on the amalgamated results of TSW, marker metabolites, mQTLs, eQTLs, and mTWAS, we have compiled a list of 30 seed size‐related candidate genes influencing *B. napus* TSW (Table S8, Supporting Information).

Additionally, through a comparative analysis of marker metabolites for SOC and TSW, we have identified 21 marker metabolites shared between SOC and TSW. These include 11 flavonoid metabolites, 1 phenylpropanoid metabolite, and 9 unknown metabolites (**Table** **1**; Figure S4, Supporting Information). Among the flavonoid metabolites, 11 exhibiting negative correlations with both SOC and TSW, are associated with 31 mQTLs (Table S2, Supporting Information). Three unknown metabolites S21_S‐0678, S21_S‐4234, and wm0031, displaying negative correlations with SOC and positive correlations with TSW, are associated with 34 mQTLs (Table S2, Supporting Information). The mQTLs mapped based on these metabolites may unveil candidate genes potentially influencing both TSW and SOC. Integrating SOC and TSW results, we observe that a flavonoid metabolite, tricin 5‐O‐hexoside (mr1083, Table 1), exhibits a correlation of r = −0.40 with SOC and r = −0.14 with TSW, while a phenylpropanoid metabolite, caffeoyl shikimic acid (mr1170, Table 1), shows a correlation of r = 0.27 with SOC and r = 0.11 with TSW.

**Table 1:** Marker metabolites for both seed oil content and 1000‐seed weight.

Metabolite	r_SOC	r_TSW	Name	Class
mr1058	−0.36	−0.15	Tricin	Flavonoids
mr1076	−0.32	−0.15	Chrysoeriol 5‐O‐hexoside	Flavonoids
mr1077	−0.41	−0.14	Chrysoeriol C‐hexoside	Flavonoids
mr1082	−0.37	−0.14	Selgin 5‐O‐hexoside	Flavonoids
mr1083	−0.40	−0.14	Tricin 5‐O‐hexoside	Flavonoids
mr1089	−0.26	−0.16	Chrysoeriol O‐malonylhexoside	Flavonoids
mr1093	−0.38	−0.14	Tricin O‐malonylhexoside	Flavonoids
mr1204	−0.35	−0.16	Chrysoeriol 7‐O‐hexoside	Flavonoids
mr1275	−0.38	−0.13	Tricin 7‐O‐hexoside	Flavonoids
mr2090	−0.28	−0.18	Phloretin	Flavonoids
S21_L‐0942	−0.36	−0.12	Isorhamnetin 3‐galactoside	Flavonoids
n00282	−0.38	−0.13	#N/A	Unknown
S21_L‐3824	−0.20	−0.12	#N/A	Unknown
S21_L‐4082	−0.21	−0.22	#N/A	Unknown
S21_L‐4831	−0.23	−0.10	#N/A	Unknown
S21_S‐0678	−0.22	0.24	#N/A	Unknown
S21_S‐1014	−0.24	−0.11	#N/A	Unknown
S21_S‐1266	0.22	0.19	#N/A	Unknown
S21_S‐4234	−0.21	0.17	#N/A	Unknown
wm0031	−0.23	0.11	#N/A	Unknown
mr1170	0.27	0.11	Caffeoyl shikimic acid	Phenylpropanoids

Referring to our ternary relationship network for SOC and TSW (Figure S4, Supporting Information), it's noteworthy that 26 mQTLs corresponding to 43 metabolites, including mr1083 and mr1170, are co‐localized on the C09 chromosome (Table S8, Supporting Information). Notably, eGWAS results for *Auxin response factor 2* (*BnaA09.ARF2*, BnaA09g05840D) coincide with mQTL1635 for mr1083, and *BnaC09.ARF2* aligns with mQTL4857 for mr1170 (Table S8, Supporting Information). The mTWAS results reveal significant associations of *BnaA09.ARF2* with mr1421 (*p* = 6.22 × 10^−06^) and S21_S‐0305 (*p* = 5.97 × 10^−06^); *BnaC09.ARF2* with wm0034 (*p* = 3.73 × 10^−06^) and S21–2486 (*p* = 7.68 × 10^−07^) (Table S3, Supporting Information). Furthermore, eGWAS of *BnaC09.ARF2* indicates several *cis*‐eQTLs on the C09 chromosome and *trans*‐eQTLs on A02, A03, A07, A09, A10, C02, C07, and C09. Similar observations are made for *BnaA09.ARF2* (Table S6, Supporting Information). ARF2, known for specifically binding to auxin response promoter elements 5′‐TGTCTC‐3′, plays a role in auxin signaling, cell division, and seed size regulation.^[^ ^65^, ^66^, ^67^ ^]^ *BnaARF2s* may impact both SOC and TSW by influencing the content of phenylpropanoid metabolites (mr1170 and mr1083), making them potential candidates for future improvements in seed traits of *B. napus*. In summary, we have identified 67 candidate genes associated with 21 marker metabolites for both SOC and TSW, presenting promising avenues for improving both *B. napus* TSW and SOC breeding (Table S9, Supporting Information).

### 2.5 *BnaA03. TGA6* is a Key Transcription Factor Gene Negatively Regulating TSW in *B. Napus*

**Figure 5:**
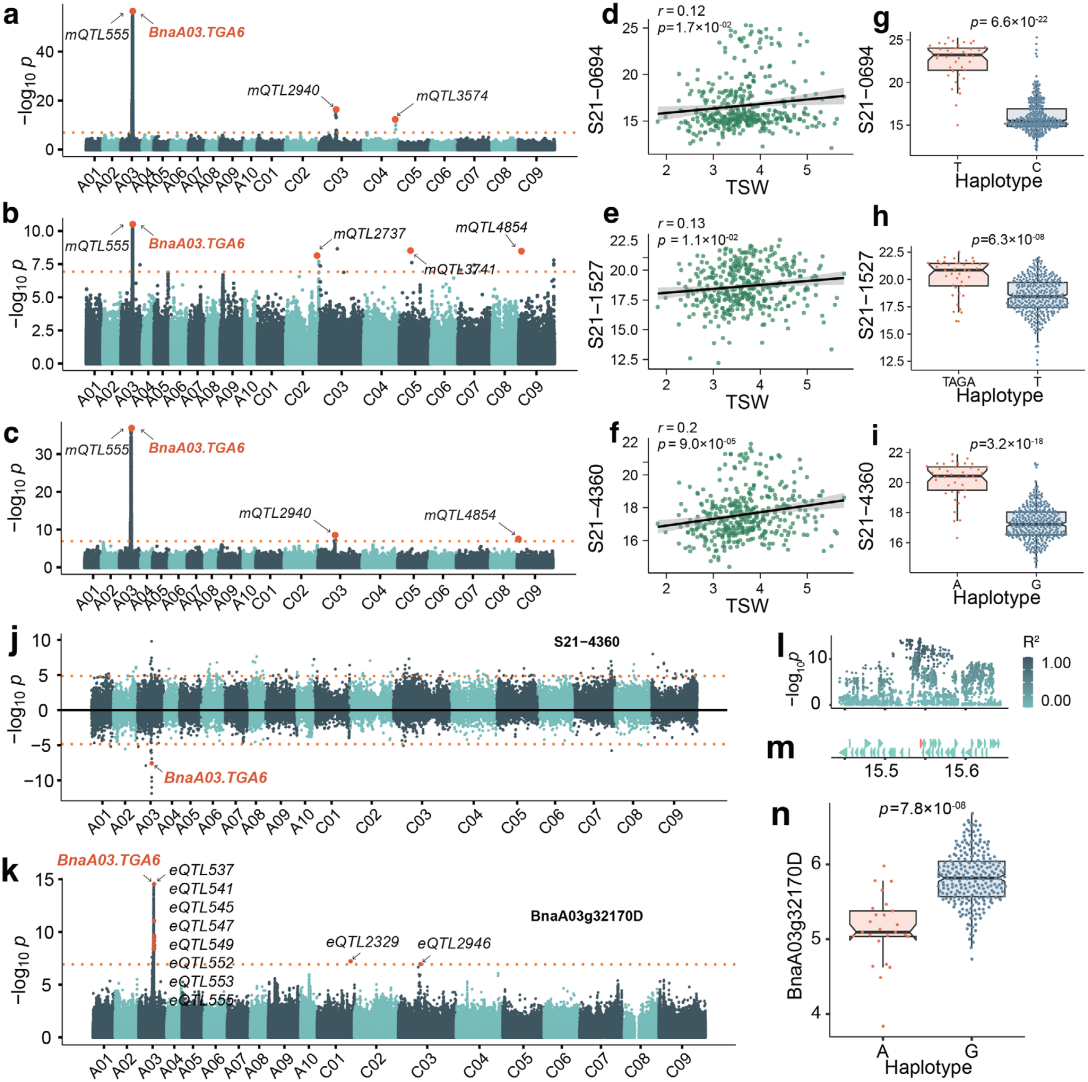
Multi‐omics analysis predicts *BnaA03.TGA6* as a regulator of TSW. a) Manhattan plot of mGWAS results (2017) of S21–0694, b) S21–1527, c) S21–4360. d) Correlation analysis of TSW and S21–0694, e) S21–1527, f) S21–4360. g) Haplotype analysis of the lead variation in *mQTL555* for S21–0694, h) S21–1527, i) S21–4360. j) Manhattan plot of mTWAS results of S21–4360. k) Manhattan plot of eGWAS results of *BnaA03.TGA6*. l,m) Local Manhattan plot of eGWAS results of *eQTL555*. n) Haplotype analysis of the lead variation in *eQTL555* for *BnaA03.TGA6*.

A notable mQTL hotspot, designated as *mQTL555* on A03, is associated with a total of 11 metabolites. All variations, position and genomic region information about the TSW correlated *mQTL555* hotspots have been summarized in Table S7 (Supporting Information). Among these, 10 metabolites S21–2077, S21–1527, S21_L‐2853, mr1169 (roseoside), S21_L‐4193, S21_S‐5484, S21–4031, S21–4360, S21–0694, and S21_S‐0841, are significantly positively correlated with TSW, while S21_S‐1322 is significantly negatively correlated with TSW (**Figure** **5**a–c; Figures S5 and S6, Supporting Information). High‐resolution spectra for these 10 unknown metabolites and mr1169 are provided (Figure S7, Supporting Information). Within these 11 unknown metabolites, three intriguing marker metabolites S21–0694, S21–1527, and S21–4360 positively correlate with TSW (Figure 5d–f). Haplotype analysis reveals that haplotype T of lead variations for S21–0694 represents higher content (Figure 5g, *p* = 6.6 × 10^−22^), haplotype TAGA represents higher content of S21–1527 (Figure 5h, *p* = 6.3 × 10^−08^), and haplotype A represents higher content of S21–4360 (Figure 5i, *p* = 3.2 × 10^−18^). In the mTWAS results, *BnaA03.TGA6* is associated with three metabolites (Figure 5j; S21–4360, *p* = 2.84 × 10^−08^; S21–4031, *p* = 4.93 × 10^−08^; S21–0694, *p* = 2.93 × 10^−11^; Table S3, Supporting Information). eGWAS analysis of *BnaA03.TGA6* reveals its association with one *cis*‐eQTL and two *trans*‐eQTLs (Figure 5k). A local Manhattan map and haplotype analysis indicate significant variation in this gene, with a *p*‐value of 7.8 × 10^−08^ (Figure 5l–n). Additionally, WGCNA analysis assigns *BnaA03.TGA6* to the turquoise module (Table S4, Supporting Information). Based on multi‐omics analysis, we posit that *BnaA03.TGA6* is a novel transcription factor affecting TSW in *B. napus*.

**Figure 6:**
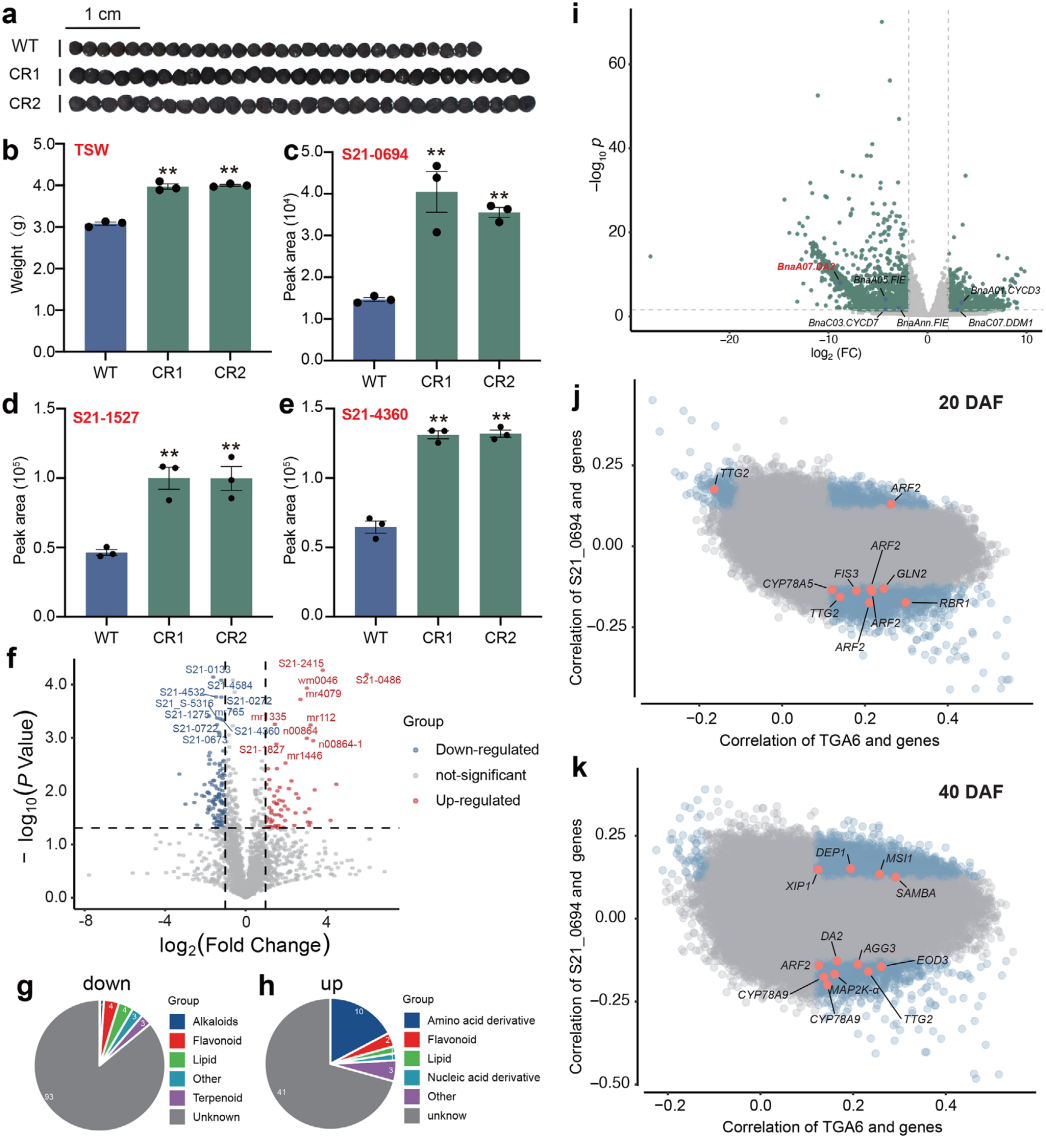
Functional identification of *BnA03.TGA6* as a negative regulator of TSW in *B. napus*. a) Seeds of CRISPR/Cas9 *TGA6 mutants* (CR1 and CR2). Bar = 1 cm. b) TSW in *TGA6 mutants* seeds and WT seeds. c) Peak area of S21–0694 d) S21–1527, e) S21–4360 in WT and *TGA6 mutants* seeds. f) The volcano plot for differentially accumulated metabolites in *TGA6 mutants* vs WT (FC > 1 and adjusted *p* < 0.05). The red and blue points indicate up‐ and down‐regulated metabolites, respectively. g,h) Number of up‐ and downregulated metabolites and their classification. i) The volcano plot for differentially expressed genes in *TGA6 mutants* vs WT of developing seed at 40 DAF (FC > 2 and adjusted *p* < 0.05). j,k) Reported TSW‐related gene expression correlation analysis with *BnaA03.TGA6* and S21–0694. The horizontal coordinates indicate the correlation between gene expression and S21–0694 at 20 and 40 DAF, and the vertical coordinates indicate the correlation between gene expression and *BnaA03.TGA6*. Blue dots indicate significantly correlated genes, and orange dots indicate reported TSW‐related genes. Values are means ± s.e.m., *n* = 3. Statistical analysis is using Student's t‐test (^*^, *p* < 0.05; ^**^, *p* < 0.01).

Examining the expression levels of *BnaTGA6* genes across various developmental stages of ZS11 seeds (https://yanglab.hzau.edu.cn/BnIR) reveals that *BnaTGA6* encompasses 6 homologous genes in ZS11. Notably, the copies on A01, A03, A05, C01, C03, and C05 exhibit similar expression patterns (Figure S8, Supporting Information). Delving into the function of *BnaA03.TGA6*, we have generated single‐gene mutants, which display no significant alterations in TSW (Figure S9, Supporting Information). Subsequently, we have created hexamutants by crossing different mutant lines of the 6 *BnaTGA6* genes in *B. napus Westar* (Table S10, Supporting Information). The TSW of two independent *BnaTGA6* hexamutant lines, CR1 and CR2, is 27.6% and 29.9% higher than that of the wild type (WT), respectively (**Figure** **6**a,b). It indicates that there is functional redundancy between *BnaA03.TGA6* and other *TGA6* genes in the genome of *B. napus*. Metabolomic analysis has unveiled significantly elevated levels of metabolites, including S21–0694 (Figure 6c), S21–1527 (Figure 6d), and S21–4360 (Figure 6e) in the mutants compared to WT. Metabolome analysis of *TGA6* mutants and WT indicates that 108 metabolites are down‐regulated, while 58 metabolites are up‐regulated (Figure 6f). Specifically, the most down‐regulated metabolites are flavonoids and lipids, while the most up‐regulated metabolites are amino acid derivatives (Figure 6g,h).

Furthermore, we have conducted RNA‐seq on developing seeds (40 DAF) of both WT and CR1. The transcriptome analysis has revealed 3715 differentially expressed genes (DEGs, log_2_FC > 2, *P‐adjust*<0.05) in CR1 compared to WT, with 1595 genes up‐regulated and 2120 down‐regulated and 6 genes related to seed size regulation exhibit significantly altered expression levels and 6 TSW related DEGs (Figure 6i). Interestingly there are two *Polycomb group protein FERTILIZATION‐INDEPENDENT ENDOSPERM* (*BnaA05.FIE* and *BnaAnn.FIE*) involved in endosperm development, are significantly decreased (Figure 6i).^[^ ^68^ ^]^ And two gene *cyclin‐D7‐1* (*BnaC03.CYCD7*) and *cyclin‐D3‐1* (*BnaA01.CYCD3*) involved in early endosperm and embryo development (Figure 6i).^[^ ^69^, ^70^ ^]^ We also found another *DDM1* (*BnaC07.DDM1*) is significantly increased in the *tga6* mutant. Interestingly, *DA2* ^[^ ^42^, ^71^ ^]^ participate in the ubiquitin pathway among the 6 TSW related genes (Figure 6i). Go enrichment of all 3715 DEGs we found that they are enriched in processes related to photosynthesis, photosystem I, and photosystem II (Figure S10, Supporting Information), indicating that the *tga6* mutant influences photosynthesis in developing seeds.

We have proved that S21–0694, S21–1527, and S21–4360 significantly increased in the *tga6* mutants (Figure 6c–e). To explore the *BnaTAG6* metabolome level mechanism, we have also provided predicted structures for these three TSW marker metabolites (Figure S11, Supporting Information). Importantly, S21_0694, the highest correlated TSW marker metabolite, has been further validated its representativeness for TSW through correlation analysis with both the population expression of *BnaA03.TGA6* and the population metabolite level of S21_0694 using transcriptome data at 20 DAF and 40 DAF. Notably, 10 reported TSW genes stand out in the 20 DAF transcriptome (Figure 6j), and 12 genes in the 40 DAF (Figure 6k). This suggests that S21_0694 is validated as a major marker for TSW, as its concentration increased in parallel with the TSW gain observed in the *tga6* mutants and associated with many TSW‐correlated genes at a population level.

### 2.6 BnaA03. TGA6 Regulates TSW by Inhibiting *BnaA07.DA2*

**Figure 7:**
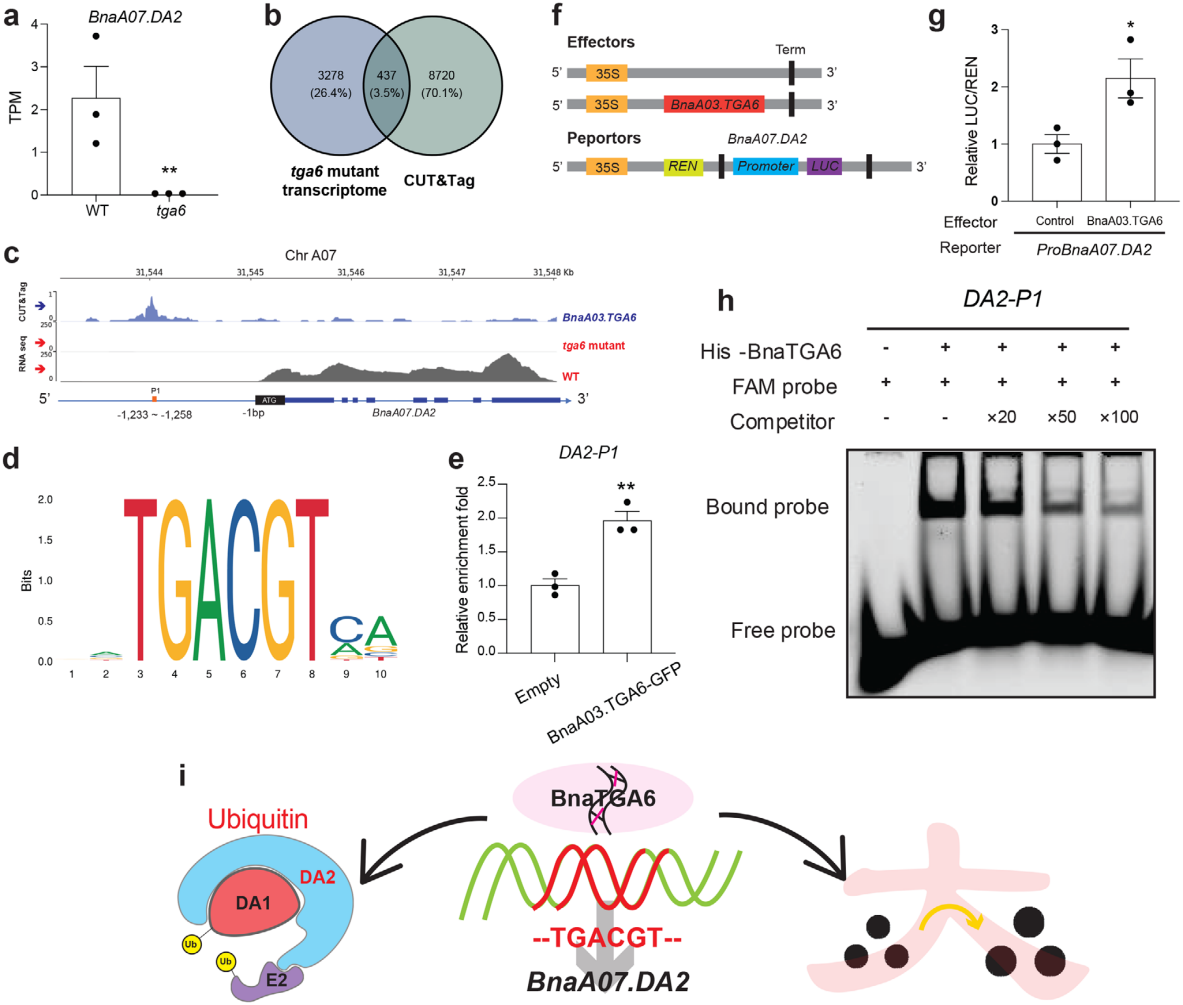
Regulatory mechanism of BnaA03.TGA6. a) Expression level of *BnaA07.DA2* in WT and *tga6* mutant developing seed (40DAF). b) Venn gram of *tga6* mutant transcriptome (log2FC > 2, *P.adj* < 0.05) and BnaA03.TGA6 CUT&Tag hits (promoter region within 2kb) c) BnaA03.TGA6 CUT&Tag binding profile and corresponding expression levels near the *BnaA07.DA2* region. Peaks are normalized to counts per million (CPM). Blue peaks represent BnaA03.TGA6 binding sites on the *BnaA07.DA2* promoter. Grey and red peaks represent expression data for *BnaA07.DA2*; red is not visible due to low expression. Orange rectangles indicate the promoter region targeted in CUT&Tag‐qPCR, shown in f. d) Enrichment of the “TGACGT” motif within the *BnaA07.DA2* promoter region targeted by BnaA03.TGA6. e) CUT&Tag‐qPCR results showing in vivo binding of BnaA03.TGA6 to the *BnaA07.DA2* promoter region. f) Schematic representation of the constructs used in the transient dual‐luciferase assay. The effector plasmid contains the *BnaA03.TGA6* coding sequence driven by the CaMV 35S promoter. The reporter plasmid includes the *REN* gene under the control of the 35S promoter and the *LUC* gene driven by the *BnaA07.DA2* promoter (*ProBnaA07.DA2*). g) Bar graph showing the relative *LUC/REN* ratio from the dual‐luciferase assay, indicating the transcriptional activation of *BnaA07.DA2* by BnaA03.TGA6. h) EMSA results demonstrating that the His‐BnaA03.TGA6 fusion protein directly binds to specific motif within the *BnaA07.DA2* promoter. i) Proposed model of transcriptional regulation: BnaTGA6 activates *BnaA07.DA2*, contributing to TSW regulation via the ubiquitin pathway. Values are means ± s.e.m. (n = 3). Statistical significance was determined by Student's t‐test (^*^, *p* < 0.05; ^**^, *p* < 0.01).

To further explore BnaTGA6 mediated regulation, we have performed a genome‐wide prediction of BnaA03.TGA6 binding motifs among the significantly altered genes and identify 1313 potential BnaA03.TGA6 interacting targets (Table S11, Supporting Information). We have found that among 6 TSW related DEGs (Figure 6i), only *BnaA07.DA2* harbored predicted BnaA03.TGA6 binding motif in its promoter region (Table S11, Supporting Information), and it also shows the significant expression fold change among the 6 DEGs (**Figure** **7**a). To detect BnaA03.TGA6 enrichment at the endogenous *BnaA07.DA2* promoter under near‐physiological chromatin conditions in vivo, we have performed Cleavage Under Targets and Tagmentation (CUT&Tag) using protoplasts isolated from rapeseed leaves. We have provided annotated sequence data in the Table S12 (Supporting Information), and there are 16390 hits in total and 8720 hits are sequenced at genes’ promoter region within 2kb. There are 437 genes are overlapped between 3715 *tga6* mutant transcriptome and 8720 BnaA03.TGA6 CUT&Tag hit genes (Figure 7b). Go enrichment analysis indicates that BnaA03.TGA6 significantly effect photosystem (Figure S12, Supporting Information). Sequencing binding profile indicates significant peaks in the *BnaA07.DA2* promoter region P1 (Figure 7c). BnaA03.TGA6 is predicted to have two types of putative binding motifs.^[^ ^72^ ^]^ Among these, the TGACGT motif, P1, is found within the *BnaA07.DA2* promoter (Figure 7d). In addition, we have adopted a targeted and sensitive CUT&Tag‐qPCR approach to quantify BnaA03.TGA6 binding at the P1 region, suggesting that BnaA03.TGA6 is significantly enriched at *Bna07.DA2* P1 promoter region (Figure 7e). To validate whether BnaA03.TGA6 regulates *BnaA07.DA2* expression, we conduct a transient dual‐luciferase assay in Arabidopsis protoplasts. The results demonstrate that *Luciferase* (*LUC*) expression is driven by the *BnaA07.DA2* promoter is significantly upregulated when co‐transformed with the BnaA03.TGA6 effector (Figure 7f,g), suggesting that BnaA03.TGA6 may activate *BnaA07.DA2* transcription. Complementarily, electrophoretic mobility shift assays (EMSAs) using purified His‐BnaA03.TGA6 protein and synthetic promoter fragments confirmed direct binding to the P1 regions of the *BnaA07.DA2* promoter in vitro (Figure 7h). Collectively, the in vivo and in vitro results demonstrate that BnaA03.TGA6 directly binds to the promoter region of *BnaA07.DA2* in the ubiquitin pathway and activates its expression, thereby negatively regulating TSW in rapeseed (Figure 7i).

## 3 Discussion

In recent years, as omics data become increasingly available, association analysis, including GWAS and TWAS, has emerged as a crucial tool for exploring genetic variations in crops.^[^ ^37^, ^38^, ^39^, ^73^, ^74^, ^75^ ^]^ Linking marker metabolites to multiple agronomic traits has identified potential breeding loci for various crops.^[^ ^6^, ^7^, ^76^ ^]^ In this study, we have identified 137 marker metabolites correlated with TSW using two years of *B. napus* mature seed metabolome and TSW data. Through association analysis (Figure 2a,b,d), we have uncovered numerous potential loci influencing *B. napus* TSW. In contrast to previous reports on genes and loci affecting *B. napus* TSW,^[^ ^51^, ^77^, ^78^, ^79^, ^80^ ^]^ our findings reveal positive correlations between amino acid derivatives and phenylpropanoid metabolites with TSW, while flavonoid metabolites exhibit a negative correlation. These results suggest the potential for improving *B. napus* TSW through metabolic pathway engineering.

Compared to limited loci from single agronomic trait linkage or association analysis, high‐throughput metabolomic data provide a rich resource for potential breeding loci.^[^ ^78^, ^81^, ^82^, ^83^ ^]^ Previous studies have identified *B. napus* other TSW loci on the A03 chromosome.^[^ ^78^, ^84^ ^]^ Such as fine mapping of *cqSW.A03‐2* narrowed the locus to a 61.6‐kb region, in which the *histidine kinase* gene BnaA03G37960D was identified as the candidate gene,^[^ ^78^ ^]^ and GWAS discovered a SNP, rs6515 on A03 (22079–22,2 kb) is associated with *B. napus* TSW.^[^ ^84^ ^]^ The discovery of *mQTL555* in this study underscores the power of combining metabolomic analysis to unveil new *B. napus* TSW loci (Figure 2a,b). But we also noticed that metabolites S21–0709 (putative flavonoid), S21–4140 (putative amino acid derivative), and S21–1134 (other) are co‐localized at mQTL1534 (Position: A08 12758480), which is co‐localized with a previous SNP (rs17921, A08, 12464–12777 kb).^[^ ^84^ ^]^ mQTL3177 (S21_L‐4233, S21‐4731, S21_L‐1836, S21‐1134; position C03 52264 kb) is co‐localized with rs17613 (Position C03 51969–52357 kb).^[^ ^84^ ^]^ These co‐localized QTLs suggest potential metabolite associations for those loci. We provided a total of 734 mQTLs in this study, most of which are not reported by earlier TSW GWAS, which provides guide information for the *B. napus* TSW breeding and needs further validation. Furthermore, TWAS offers unique advantages over GWAS by associating single genes with traits rather than linkage intervals.^[^ ^35^, ^36^, ^37^, ^38^, ^39^ ^]^ Our comprehensive mTWAS of *B. napus* TSW correlated metabolites furnishes extensive genetic evidence and candidate genes potentially influencing *B. napus* TSW (Table S3, Supporting Information). Notably, based on multi‐omics data, we have constructed the first TSW triple relationship network (metabolite‐gene‐QTL) for *B. napus* (Figure 4), laying a metabonomic foundation for future *B. napus* TSW breeding.

The identification of *BnaC09.DDM1*, an ATP‐dependent chromatin remodeler essential for maintaining DNA methylation, suggests a potential mechanistic link between epigenetic regulation and metabolic control during seed development. Our findings imply that it may also influence seed metabolite composition, thereby indirectly contributing to agronomic traits such as TSW. On the other hand, through multi‐omics analysis, we have identified a novel gene, *BnaA03.TGA6*. Considering gene function redundancy, we have generated hexamutants of *BnaTGA6* (Figure S9, Supporting Information). The seeds of the *TGA6* mutant materials are significantly larger than the WT (Figure 5a,b). Interestingly, three out of eleven metabolites positively correlated with TSW are associated with the *BnaA03.TGA6* locus, and their metabolite levels changed accordingly (Figure 6c–e). This suggests that alterations in *B. napus* TSW are accompanied by changes in metabolite content. Whole‐genome scanning reveals more metabolites associated with *mQTL555*, with the majority being annotated as flavonoids and phenylpropanoid metabolites (Table S12, Supporting Information). Given the tight association between mQTL and metabolites, those metabolites having the same mQTL may share structural similarities. It's possible that *BnaA03.TGA6* may also participate in flavonoid and phenylpropanoid biosynthesis regulation, or there are more genes clustered at *mQTL555* involved in the biosynthesis pathways. We have also analyzed the correlation between 20 DAF and 40 DAF with the expression profile of *BnaA03.TGA6* (Figure 6j,k) and emphasize the potential of S21_0694 been a valuable TSW marker metabolite. For *BnaA03.TGA6* regulation validation, we have performed dual‐luciferase assay, CUT&Tag, and EMSA with *BnaA07.DA2*. Combining *TGA6* mutant transcriptional data, we find that *BnaA07.DA2* is the downstream target of *BnaA03.TGA6*, activated by it (Figure 7). In addition, we have fully investigated the *tga6* 40DAF transcriptome data, find that *BnaA09.MET1* ^[^ ^50^ ^]^ is significantly increased in the *tga6* mutant (Figure S13a, Supporting Information) and predicted with 1 binding motif of BnaA03.TGA6 (Figure S13b, Supporting Information). However, dual‐luciferase assay shows that *BnaA09.MET1* is activated by BnaA03.TGA6 (Figure S13c, Supporting Information) and not identified by CUT&Tag. It suggests that *BnaA09.MET1* is not the downstream target of BnaA03.TGA6. We also thoroughly investigate the BnaA03.TGA6 CUT&Tag sequencing data, find that BnaA03.TGA6 is enriched at the promoters’ region of *BnaA01.TTG2* ^[^ ^54^ ^]^ and *BnaC01.TOP1‐α* ^[^ ^54^ ^]^ within 1kb (Table S12, Supporting Information). And *BnaA01.TTG2* is predicted with 1 binding motif of BnaA03.TGA6 and *BnaC01.TOP1‐α* with 4 (Figure S13b, Supporting Information). Dual‐luciferase assay shows that *BnaC01.TOP1‐α* is repressed by BnaA03.TGA6 and *BnaA01.TTG2* is activated by BnaA03.TGA6 (Figure S13d,e, Supporting Information). Interestingly, BnaA03.TGA6 is enriched at the promoter region of 4 *BnaDA1* ^[^ ^42^ ^]^ homologue genes (Table S12, Supporting Information) but not significantly changed at *tga6* 40DAF transcriptome. *BnaA08.DA1* and *BnaA08.DA1* are both predicted with 5 binding motifs of BnaA03.TGA6 and *BnaA06.DA1* and *BnaC05.DA1* don't have BnaA03.TGA6 binding motifs (Figure S13b, Supporting Information). Then, we have chosen *BnaA08.DA1* to perform validation experiment by CUT&Tag‐qPCR and EMSA. Only two BnaA03.TGA6 binding motifs at the promoter region of *BnaA08.DA1* are showing interaction (Figure S13f,g, Supporting Information). We believe that BnaA03.TGA6 is interacting with multiple downstream targets within different pathways, especially *BnaA08.DA1* and *BnaA07.DA2* of ubiquitin pathway at different seed developing stages. And BnaA03.TGA6 is binding *BnaA07.DA2* promoter region P1 to control TSW at 40DAF.

We consider this an exemplary instance of discovering novel genes for crop agronomic traits, building on our earlier work on SOC.^[^ ^12^ ^]^ Through multi‐omics analysis, we have identified and confirmed the impact of *BnaA03.TGA6* on TSW, demonstrating its ability to activate *BnaA07.DA2* expression. Our findings contribute to the understanding of the regulatory mechanisms governing seed size in plants. *BnaA03.TGA6*, a transcription factor of the b‐ZIP superfamily and part of the second branch of TGA in Arabidopsis, is known for its involvement in salicylic acid reactions and plant immunity.^[^ ^60^, ^61^, ^62^, ^63^ ^]^ Prior research on *TGA6* has primarily focused on plant immune responses; this study discovered for the first time its new function in rapeseed seed development, suggesting the differentiation of gene function in evolution.

Due to the extraction method, our study has concentrated on water‐soluble metabolites, known for their high confidence.^[^ ^16^, ^17^, ^18^ ^]^ While our analysis spanned two years, the significant variance in metabolites correlated to TSW between 2017 and 2018 led us to intersect the data for subsequent analysis (Figure 1a,b). The 137 TSW‐correlated metabolites demonstrated good repeatability (Figure 1c), mitigating systematic errors to a certain extent. Most of these markers are unknown metabolites, necessitating further investigation. Techniques involving genetic analysis and mass spectrum ion information can aid in inferring the structure of unknown metabolites.^[^ ^16^ ^]^ Additionally, algorithm analysis, including machine learning and artificial intelligence, has been proposed to assist in metabolite annotation and structure prediction, potentially expediting metabolomics research.^[^ ^85^, ^86^, ^87^ ^]^ Although the functional annotation of unknown metabolites still needs to be further explored, the metabolite‐QTL network provided by this study lays a foundation for the development of molecular markers.

## 4 Experimental Section

### Plant Materials, Collection of Phenotype Data

Based on the previous study, 388 accessions were selected from a population containing 505 accessions that were grown in Wuhan (2017, 2018).^[^ ^37^ ^]^ The WSeen SC‐G TSW analysis system (Hangzhou WSeen Detection Technology Co., Ltd., China) was used to acquire TSW phenotype data, 6 replicates for each accession. Transgenic *B. napus* materials were arranged in the transgenic crop field at Huazhong Agricultural University. Randomized complete block designs were applied for the field trial with three replications. Seed metabolome data used in this study were obtained from Li et al. (2023),^[^ ^12^ ^]^ in which 2172 metabolites were obtained. All metabolite extraction and data analysis procedures were performed as described in the original publication.^[^ ^12^ ^]^

### mGWAS, mTWAS, eGWAS, and Co‐Expression Analysis

The variants used for mGWAS and eGWAS were called by a meta‐analysis of reads.^[^ ^37^, ^88^ ^]^ *B. napus* genome was used as the reference genome (*B. napus* v4.1, http://www.genoscope.cns.fr/brassicanapus/). Variations filtered with minor allele frequency > 5% were collected with PLINK.^[^ ^89^ ^]^ TSW‐correlated metabolites content in 2017 and 2018 (8274830), and gene expressions (8258336) were performed using mGWAS and eGWAS by FaST‐LMM software.^[^ ^90^ ^]^ The threshold was set at Bonferroni correction of *p* = 1.2 × 10^−07^, and Manhattan plots were generated using the R package. Similarly, 274 accessions transcriptome data at 40 DAF (2017, Wuhan) were used as described before.^[^ ^37^ ^]^ Using linear regression, mTWAS was performed within 2017 TSW‐correlated metabolites and gene expression, and the threshold was set at 1/70781.^[^ ^91^ ^]^ Then, these genes were then used to conduct co‐expression analysis with an R package (WGCNA).^[^ ^92^ ^]^ Finally, 139 modules were clustered, and the scale‐free topology of the determined networks was approximated based on a soft‐thresholding power (β = 6).

### Network Building

For each QTL, the edges between TSW‐correlated metabolites were generated based on the significant signals of mQTLs; likewise, the edges between genes were based on the significant signals of eQTLs. In detail, all the significant signals over the threshold for 137 TSW‐correlated metabolites were clustered into mQTL LD blocks (100 kb). On the other hand, 9077 genes related to the 137 TSW‐correlated metabolites (*p* < 1.41 × 10^−5^) were selected for conducting eGWAS.^[^ ^91^ ^]^ All the significant signals over the threshold were clustered into eQTL LD blocks (100 kb). Finally, both mQTLs and eQTLs distance was calculated; if the distance was within 100 kb, it was considerd that there was a connection among TSW‐correlated metabolite, gene, and QTL (representing both mQTL and eQTL).

### Vector Construction and Plant Transformation

The CRISPR‐Cas9 genome editing system^[^ ^93^ ^]^ was used to create *TGA6* mutant materials. Golden Gate Assembly was used to conduct the transformation *pKSE401* vector with the PCR fragment. Then, the recombinant vector was used to create TGA6 mutant materials. For detailed methods, please refer to Dai et al.^[^ ^94^ ^]^ *BnaTGA6* hexamutants were created by crossing different mutant lines, and sequencing primers for *TGA6* mutant materials were listed, which were used to confirm the mutant types (Table S14, Supporting Information).

### Metabolite Extraction

A total of 0.1 g of mature *B. napus* seeds for WT and *tga6* mutant was collected for each sample and homogenized in 1 mL of 70% methanol containing 0.1 mg L^−1^ acyclovir (internal standard) using a TissueLyser II (Qiagen, Germany) at 29 Hz for 60 seconds. The homogenate was vortexed and incubated at 4 °C for 10 h to extract metabolites. Following centrifugation at 9000 × g for 10 min, the supernatants were collected, pooled, and filtered through a 0.22 µm organic membrane filter (SCAA‐104; ANPEL, Shanghai, China) prior to LC‐MS analysis.

### Dual‐Luciferase Assay

The Dual‐Luciferase Reporter Assay System (Promega, Madison, WI, USA) was utilized to perform dual‐luciferase assays. To construct the reporter plasmid, the promoters for *BnaA09.MET1*, *BnaC01.TOP1‐α*, *BnaA01.TTG2* and *BnaA07.DA2* was amplified and inserted into the pGreenII0800‐LUC vector. Similarly, the effecter plasmids were created by amplifying and inserting the open reading frame (ORF) of *BnaA03.TGA6* into the pM999‐YFP vector. The experimental procedure was conducted in Arabidopsis protoplasts as described by Yoo et al.^[^ ^95^ ^]^ After 16 h, the transformed Arabidopsis protoplasts were lysed in 100 µL of passive lysate. Following a 30 s incubation, luminescence measurements were carried out using the SPARKRMULTIMODE MICROPLATE (TECAN, Swiss) with the subsequent steps: 50 µL of the firefly luciferase reagent (LARII) was added to the test sample, followed by a 10 s equilibration period and luminescence measurement (10 s integration time). This was followed by the addition of 50 µL of the REN reagent and firefly quenching (Stop and Glow TM buffer), another 10 s equilibration period, and luminescence measurement (10 s integration time). The data were then presented as the ratio of firefly to Renilla luciferase activity (Fluc/Rluc). Each data point was obtained from a minimum of three biological replicates, and a total of 15 repeats were performed for each assay to ensure statistical reliability and accuracy. Primers were designed using NCBI Primer‐BLAST and were listed in Table S14 (Supporting Information).

### RNA Extraction and RNA‐seq

Developing seeds from wild‐type (WT) and mutant plants grown in Wuhan were subjected to RNA‐seq analysis. Seeds at 40 days after flowering (DAF) were collected, with three biological replicates per genotype. Detailed sampling procedures were described previously.^[^ ^37^ ^]^ Total RNA was extracted using the RNAprep Pure Plant Kit (TIANGEN, China). RNA‐seq libraries were prepared with the NEBNext Ultra RNA Library Prep Kit for Illumina (NEB, USA; Cat. #E7530L) and sequenced on an Illumina HiSeq 4000 platform.

Quality control of sequencing data was performed using MultiQC,^[^ ^96^ ^]^ and transcript quantification was conducted with STAR^[^ ^97^ ^]^ against the Darmor v5 reference genome annotation. Gene expression levels were estimated using the RSEM.^[^ ^98^ ^]^ Differential gene expression analysis was performed with the R package DESeq2.^[^ ^99^ ^]^

### Protoplast Isolation, CUT&Tag Assay, and CUT&Tag‐qPCR

Transient transformation of rapeseed leaf protoplasts was performed for CUT&Tag assays using the Hyperactive Universal CUT&Tag Assay Kit (Vazyme, TD904). Rapeseed protoplast isolation was adapted from the established protocol for Arabidopsis leaf protoplast extraction.^[^ ^95^ ^]^ The full‐length coding sequence of *BnaA03.TGA6* was cloned into the pM999‐GFP expression vector under the control of the CaMV 35S promoter via the XbaI restriction site. The recombinant plasmid was introduced into rapeseed protoplasts by polyethylene glycol (PEG)‐mediated transformation. After incubation for 16 h, protoplasts exhibiting GFP fluorescence were collected.

The CUT&Tag sequencing data analysis method is adapted from a previous paper.^[^ ^100^ ^]^ In short, the successfully constructed libraries were subjected to high‐throughput sequencing on the Illumina NovaSeq platform by Novogene (Beijing, China). Clean reads were aligned to the ZS11 v0 reference genome using BWA (v0.7.17) with the MEM algorithm.^[^ ^101^ ^]^ Peak calling was performed with high confidence using MACS2 (v2.2.7.1) under the parameters “–gsize 1e9 –tsize 150 ‐B –nomodel –shift 100 –extsize 200 –qvalue 0.01 ‐f BAMPE”.^[^ ^102^ ^]^ Peak regions were visualized with pyGenomeTracks.^[^ ^103^ ^]^ Motif analysis was conducted using FIMO and meme‐chip from the MEME Suite.^[^ ^104^ ^]^ Immunoprecipitated DNA was subjected to CUT&Tag‐qPCR to validate the enrichment of target regions, using SYBR Premix TransStart Green qPCR SuperMix (TransGen, China) on a Bio‐Rad CFX Connect Real‐Time PCR System. A spike‐in DNA was included as a negative control. Primers for CUT&Tag‐qPCR validation were designed based on identified motifs and were listed in Table S14 (Supporting Information).

### RT‐qPCR

RT‐qPCR was performed using SYBR Green chemistry on a CFX Connect Real‐Time PCR System (Bio‐Rad, USA). Gene expression levels were normalized to ACTIN7 (BnaA06G0089200WE). Reactions were carried out in 96‐well Hard‐Shell PCR plates (Bio‐Rad, USA) using 0.4 µm gene‐specific primers and SYBR Premix TransStart Green qPCR SuperMix (TransGen, China) in a final volume of 15 µL. Each sample included three biological replicates and three technical replicates. The thermal cycling conditions were: initial denaturation at 95 °C for 1 min, followed by 45 cycles of 95 °C for 15 s, 60 °C for 15 s, and 72 °C for 20 s. Primers were designed using NCBI Primer‐BLAST and were listed in Table S14 (Supporting Information).

### Electrophoretic Mobility Shift Assay (EMSA)

The full‐length coding sequence of *BnaA03.TGA6* was amplified from ZS11 cDNA and cloned into the pET15D vector. The resulting His‐BnaA03.TGA6 fusion protein was expressed in *Escherichia coli Rosetta* cells induced with 0.4 mM IPTG at 16 °C for 16 h. The fusion protein was purified by NTA‐Ni.

Promoter fragments of *BnaA08.DA1* and *BnaA07.DA2*, containing predicted binding motifs, was synthesized and labeled with FAM at the 5′ end. Forward and reverse oligonucleotides were annealed by heating at 98 °C for 10 min, followed by gradual cooling to room temperature to form double‐stranded probes. Both FAM‐labeled and unlabeled probes were incubated with purified His‐BnaA03.TGA6 protein in EMSA/Gel‐shift Binding Buffer (Beyotime, GS005) at 23 °C for 30 min.

Binding reactions were resolved on a 6% (w/v) native polyacrylamide gel at 80 V for 1 h in 0.5× TBE buffer (45 mm Tris‐base, 45 mm boric acid, 0.5 mm EDTA, pH 8.3) at 4 °C in the dark. Fluorescent signals from FAM‐labeled probes were detected using an Amersham Typhoon imaging system (Cytiva). All oligonucleotide sequences used for EMSA were listed in Table S14 (Supporting Information).

## Conflict of Interest

The authors declare no conflict of interest.

## Author Contributions

L.L., Z.T., and X.W. contributed equally to this work. L.G., W.C., and X.Y. designed and supervised this study. L.L., X.W., and J.C. performed the experiments. Z.T., Z.T., H.Z., X.H., Y.X., and L.L. analyzed the data. L.L. and Z.T. wrote the manuscript. L.G., W.C., and X.Y. revised the manuscript. All authors read and approved the manuscript.

## Supporting information



Supporting Information

Supplemental Table 1

## Data Availability

The data that support the findings of this study are available from the corresponding author upon reasonable request.

## References

[1] A. R. Fernie , R. N. Trethewey , A. J. Krotzky , L. Willmitzer , Nat. Rev. Mol. Cell Biol. 2004, 5, 763. 15340383 10.1038/nrm1451

[2] J. Venegas‐Molina , F. J. Molina‐Hidalgo , E. Clicque , A. Goossens , Trends Plant Sci. 2021, 26, 472. 33478816 10.1016/j.tplants.2020.12.008

[3] R. C. Meyer , M. Steinfath , J. Lisec , M. Becher , H. Witucka‐Wall , O. Törjék , O. Fiehn , Ä. Eckardt , L. Willmitzer , J. Selbig , T. Altmann , Proc. Natl. Acad. Sci. USA 2007, 104, 4759. 17360597 10.1073/pnas.0609709104 PMC1810331

[4] R. Sulpice , E.‐T. Pyl , H. Ishihara , S. Trenkamp , M. Steinfath , H. Witucka‐Wall , Y. Gibon , B. Usadel , F. Poree , M. C. O. Piques , M. Von Korff , M. C. Steinhauser , J. J. B. Keurentjes , M. Guenther , M. Hoehne , J. Selbig , A. R. Fernie , T. Altmann , M. Stitt , Proc. Natl. Acad. Sci. USA 2009, 106, 10348. 19506259 10.1073/pnas.0903478106 PMC2693182

[5] S. Bijlsma , I. Bobeldijk , E. R. Verheij , R. Ramaker , S. Kochhar , I. A. Macdonald , B. van Ommen , A. K. Smilde , Anal. Chem. 2006, 78, 567. 16408941 10.1021/ac051495j

[6] N. Carreno‐Quintero , A. Acharjee , C. Maliepaard , C. W. B. Bachem , R. Mumm , H. Bouwmeester , R. G. F. Visser , J. J. B. Keurentjes , Plant Physiol. 2012, 158, 1306. 22223596 10.1104/pp.111.188441 PMC3291263

[7] P. T. Do , M. Prudent , R. Sulpice , M. Causse , A. R. Fernie , Plant Physiol. 2010, 154, 1128. 20841452 10.1104/pp.110.163030 PMC2971594

[8] T. Obata , A. R. Fernie , Cell. Mol. Life Sci. 2012, 69, 3225. 22885821 10.1007/s00018-012-1091-5 PMC3437017

[9] R. Sulpice , P. C. McKeown , Annu. Rev. Plant. Biol. 2015, 66, 187. 25621519 10.1146/annurev-arplant-043014-114720

[10] C. Riedelsheimer , J. Lisec , A. Czedik‐Eysenberg , R. Sulpice , A. Flis , C. Grieder , T. Altmann , M. Stitt , L. Willmitzer , A. E. Melchinger , Proc. Natl. Acad. Sci. USA 2012, 109, 8872. 22615396 10.1073/pnas.1120813109 PMC3384205

[11] J. Wang , P. Zhou , X. Shi , N. Yang , L. Yan , Q. Zhao , C. Yang , Y. Guan , Crop J. 2019, 7, 651.

[12] L. Li , Z. Tian , J. Chen , Z. Tan , Y. Zhang , H. Zhao , X. Wu , X. Yao , W. Wen , W. Chen , L. Guo , Genome Biol. 2023, 24, 141. 37337206 10.1186/s13059-023-02984-z PMC10278308

[13] A. R. Fernie , T. Tohge , Annu. Rev. Genet. 2017, 51, 287. 28876980 10.1146/annurev-genet-120116-024640

[14] M. Zaynab , M. Fatima , S. Abbas , Y. Sharif , M. Umair , M. H. Zafar , K. Bahadar , Microb. Pathog. 2018, 124, 198. 30145251 10.1016/j.micpath.2018.08.034

[15] W. Chen , L. Gong , Z. Guo , W. Wang , H. Zhang , X. Liu , S. Yu , L. Xiong , J. Luo , Mol. Plant 2013, 6, 1769. 23702596 10.1093/mp/sst080

[16] W. Chen , Y. Gao , W. Xie , L. Gong , K. Lu , W. Wang , Y. Li , X. Liu , H. Zhang , H. Dong , W. Zhang , L. Zhang , S. Yu , G. Wang , X. Lian , J. Luo , Nat. Genet. 2014, 46, 714. 24908251 10.1038/ng.3007

[17] W. Wen , D. Li , X. Li , Y. Gao , W. Li , H. Li , J. Liu , H. Liu , W. Chen , J. Luo , J. Yan , Nat. Commun. 2014, 5, 3438. 24633423 10.1038/ncomms4438 PMC3959190

[18] G. Zhu , S. Wang , Z. Huang , S. Zhang , Q. Liao , C. Zhang , T. Lin , M. Qin , M. Peng , C. Yang , X. Cao , X. Han , X. Wang , E. van der Knaap , Z. Zhang , X. Cui , H. Klee , A. R. Fernie , J. Luo , S. Huang , Cell 2018, 172, 249. 29328914 10.1016/j.cell.2017.12.019

[19] L. C. Solberg Woods , Physiol. Genomics 2014, 46, 81. 24326347 10.1152/physiolgenomics.00127.2013 PMC4073892

[20] T. M. Jamann , P. J. Balint‐Kurti , J. B. Holland , Methods Mol. Biol. 2015, 1284, 257. 25757777 10.1007/978-1-4939-2444-8_13

[21] J. Liu , W. Hua , Z. Hu , H. Yang , L. Zhang , R. Li , L. Deng , X. Sun , X. Wang , H. Wang , Proc. Natl. Acad. Sci. USA 2015, 112, E5123. 26324896 10.1073/pnas.1502160112 PMC4577148

[22] L. Shi , J. Song , C. Guo , B. Wang , Z. Guan , P. Yang , X. Chen , Q. Zhang , G. J. King , J. Wang , K. Liu , Plant J. 2019, 98, 524. 30664290 10.1111/tpj.14236

[23] Z. Hu , S.‐J. Lu , M.‐J. Wang , H. He , L. Sun , H. Wang , X.‐H. Liu , L. Jiang , J.‐L. Sun , X. Xin , W. Kong , C. Chu , H.‐W. Xue , J. Yang , X. Luo , J.‐X. Liu , Mol. Plant 2018, 11, 736. 29567449 10.1016/j.molp.2018.03.005

[24] A. Abdellaoui , L. Yengo , K. J. H. Verweij , P. M. Visscher , Am. J. Hum. Genet. 2023, 110, 179. 36634672 10.1016/j.ajhg.2022.12.011 PMC9943775

[25] V. Tam , N. Patel , M. Turcotte , Y. Bossé , Nat. Rev. Genet. 2019, 20, 467. 31068683 10.1038/s41576-019-0127-1

[26] Q. Di , L. Dong , L. Jiang , X. Liu , P. Cheng , B. Liu , G. Yu , Front. Plant. Sci. 2023, 14, 1268511. 38046612 10.3389/fpls.2023.1268511 PMC10691256

[27] J. Wang , B. Jiao , C. Qu , C. Yan , D. Shi , C. Jiang , M. Yuan , W. Wang , C. Yuan , X. Zhao , Q. Sun , Y. Mou , Q. Wang , Y. Li , C. Li , S. Shan , Theor. Appl. Genet. 2025, 138, 144. 40494995 10.1007/s00122-025-04923-x

[28] C. Zhan , L. Lei , Z. Liu , S. Zhou , C. Yang , X. Zhu , H. Guo , F. Zhang , M. Peng , M. Zhang , Y. Li , Z. Yang , Y. Sun , Y. Shi , K. Li , L. Liu , S. Shen , X. Wang , J. Shao , X. Jing , Z. Wang , Y. Li , T. Czechowski , M. Hasegawa , I. Graham , T. Tohge , L. Qu , X. Liu , A. R. Fernie , L.‐L. Chen , et al., Nat. Plants. 2020, 6, 1447. 33299150 10.1038/s41477-020-00816-7

[29] W. Wen , K. Li , S. Alseekh , N. Omranian , Plant Cell 2015, 27, 1839. 26187921 10.1105/tpc.15.00208 PMC4531352

[30] J. Chen , X. Hu , T. Shi , H. Yin , D. Sun , Y. Hao , X. Xia , J. Luo , A. R. Fernie , Z. He , W. Chen , Plant Biotechnol. J. 2020, 18, 1722. 31930656 10.1111/pbi.13335 PMC7336285

[31] W. Chen , L. Gong , Z. Guo , W. Wang , H. Zhang , X. Liu , S. Yu , L. Xiong , J. Luo , Mol. Plant 2013, 6, 1769. 23702596 10.1093/mp/sst080

[32] K. Cao , B. Wang , W. Fang , G. Zhu , C. Chen , X. Wang , Y. Li , J. Wu , T. Tang , Z. Fei , J. Luo , L. Wang , Genome Biol. 2022, 23, 146. 35788225 10.1186/s13059-022-02719-6 PMC9254577

[33] S. Song , L. Zhang , Y. Zhao , C. Sheng , W. Zhou , S. S. K. Dossou , L. Wang , J. You , R. Zhou , X. Wei , X. Zhang , Plant J. 2022, 112, 1051. 36176211 10.1111/tpj.15995

[34] W. Zhang , S. Alseekh , X. Zhu , Q. Zhang , A. R. Fernie , H. Kuang , W. Wen , Plant J. 2020, 104, 613. 32772408 10.1111/tpj.14950

[35] A. Gusev , A. Ko , H. Shi , G. Bhatia , W. Chung , B. W. J. H. Penninx , R. Jansen , E. J. C. de Geus , D. I. Boomsma , F. A. Wright , P. F. Sullivan , E. Nikkola , M. Alvarez , M. Civelek , A. J. Lusis , T. Lehtimäki , E. Raitoharju , M. Kähönen , I. Seppälä , O. T. Raitakari , J. Kuusisto , M. Laakso , A. L. Price , P. Pajukanta , B. Pasaniuc , Nat. Genet. 2016, 48, 245. 26854917 10.1038/ng.3506 PMC4767558

[36] A. Gusev , N. Mancuso , H. Won , M. Kousi , H. K. Finucane , Y. Reshef , L. Song , A. Safi , S. McCarroll , B. M. Neale , R. A. Ophoff , M. C. O'Donovan , G. E. Crawford , D. H. Geschwind , N. Katsanis , P. F. Sullivan , B. Pasaniuc , A. L. Price , Nat. Genet. 2018, 50, 538. 29632383 10.1038/s41588-018-0092-1 PMC5942893

[37] S. Tang , H. Zhao , S. Lu , L. Yu , G. Zhang , Y. Zhang , Q.‐Y. Yang , Y. Zhou , X. Wang , W. Ma , W. Xie , L. Guo , Mol. Plant 2021, 14, 470. 33309900 10.1016/j.molp.2020.12.003

[38] Z. Li , P. Wang , C. You , J. Yu , X. Zhang , F. Yan , Z. Ye , C. Shen , B. Li , K. Guo , N. Liu , G. N. Thyssen , D. D. Fang , K. Lindsey , X. Zhang , M. Wang , L. Tu , New. Phytol. 2020, 226, 1738. 32017125 10.1111/nph.16468

[39] Y. Ma , L. Min , J. Wang , Y. Li , Y. Wu , Q. Hu , Y. Ding , M. Wang , Y. Liang , Z. Gong , S. Xie , X. Su , C. Wang , Y. Zhao , Q. Fang , Y. Li , H. Chi , M. Chen , A. H. Khan , K. Lindsey , L. Zhu , X. Li , X. Zhang , New. Phytol. 2021, 231, 165. 33665819 10.1111/nph.17325 PMC8252431

[40] E. E. Schadt , S. A. Monks , T. A. Drake , A. J. Lusis , N. Che , V. Colinayo , T. G. Ruff , S. B. Milligan , J. R. Lamb , G. Cavet , P. S. Linsley , M. Mao , R. B. Stoughton , S. H. Friend , Nature 2003, 422, 297. 12646919 10.1038/nature01434

[41] J. Clarke , G. Simpson , Can J. Plant. Sci. 1978, 58, 731.

[42] T. Xia , N. Li , J. Dumenil , J. Li , A. Kamenski , M. W. Bevan , F. Gao , Y. Li , Plant Cell. 2013, 25, 3347. 24045020 10.1105/tpc.113.115063 PMC3809536

[43] S. Li , Y. Liu , L. Zheng , L. Chen , N. Li , F. Corke , Y. Lu , X. Fu , Z. Zhu , M. W. Bevan , Y. Li , New. Phytol. 2012, 194, 690. 22380792 10.1111/j.1469-8137.2012.04083.x

[44] D. Chakravorty , Y. Trusov , W. Zhang , B. R. Acharya , M. B. Sheahan , D. W. McCurdy , S. M. Assmann , J. R. Botella , Plant J. 2011, 67, 840. 21575088 10.1111/j.1365-313X.2011.04638.x

[45] R. Xu , H. Yu , J. Wang , P. Duan , B. Zhang , J. Li , Y. Li , J. Xu , J. Lyu , N. Li , T. Chai , Y. Li , Plant J. 2018, 95, 937. 29775492 10.1111/tpj.13971

[46] M. Zhang , H. Wu , J. Su , H. Wang , Q. Zhu , Y. Liu , J. Xu , W. Lukowitz , S. Zhang , Plant J. 2017, 92, 1005. 29024034 10.1111/tpj.13737

[47] T. Guo , K. Chen , N.‐Q. Dong , C.‐L. Shi , W.‐W. Ye , J.‐P. Gao , J.‐X. Shan , H.‐X. Lin , Plant Cell 2018, 30, 871. 29588389 10.1105/tpc.17.00959 PMC5973843

[48] R. Xu , P. Duan , H. Yu , Z. Zhou , B. Zhang , R. Wang , J. Li , G. Zhang , S. Zhuang , J. Lyu , N. Li , T. Chai , Z. Tian , S. Yao , Y. Li , Mol. Plant 2018, 11, 860. 29702261 10.1016/j.molp.2018.04.004

[49] S. Liu , L. Hua , S. Dong , H. Chen , X. Zhu , J. Jiang , F. Zhang , Y. Li , X. Fang , F. Chen , Plant J. 2015, 84, 672. 26366992 10.1111/tpj.13025

[50] W. Xiao , R. C. Brown , B. E. Lemmon , J. J. Harada , R. B. Goldberg , R. L. Fischer , Plant Physiol. 2006, 142, 1160. 17012404 10.1104/pp.106.088849 PMC1630758

[51] J.‐L. Wang , M.‐Q. Tang , S. Chen , X.‐F. Zheng , H.‐X. Mo , S.‐J. Li , Z. Wang , K.‐M Zhu , L.‐N. Ding , S.‐Y. Liu , Y.‐H. Li , X.‐L. Tan , Plant. Biotechnol. J. 2017, 15, 1024. 28097785 10.1111/pbi.12696 PMC5506660

[52] N. Li , R. Xu , Y. Li , Annu. Rev. Plant Biol. 2019, 70, 435. 30795704 10.1146/annurev-arplant-050718-095851

[53] N. Li , Y. Li , J. Exp. Bot. 2015, 66, 1087. 25609830 10.1093/jxb/eru549

[54] C. Li , X. Gong , B. Zhang , Z. Liang , C. E. Wong , B. Y. H. See , H. Yu , PLoS Biol. 2020, 18, 3000930. 10.1371/journal.pbio.3000930 PMC7673560 33156841

[55] D. Garcia , J. N. Fitz Gerald , F. Berger , Plant Cell. 2005, 17, 52. 15598800 10.1105/tpc.104.027136 PMC544489

[56] F. Chen , W. Lin , W. Li , J. Hu , Z. Li , L. Shi , Z. Zhang , Y. Xiu , S. Lin , BMC Plant Biol. 2023, 23, 268. 37208597 10.1186/s12870-023-04267-y PMC10197815

[57] C. Miller , R. Wells , N. McKenzie , M. Trick , J. Ball , A. Fatihi , B. Dubreucq , T. Chardot , L. Lepiniec , M. W. Bevan , Plant Cell. 2019, 31, 2370. 31439805 10.1105/tpc.18.00577 PMC6790077

[58] L. Ge , J. Yu , H. Wang , D. Luth , G. Bai , K. Wang , R. Chen , Proc. Natl. Acad. Sci. USA 2016, 113, 12414. 27791139 10.1073/pnas.1611763113 PMC5098654

[59] H.‐W. Wang , B. Zhang , Y.‐J. Hao , J. Huang , A.‐G. Tian , Y. Liao , J.‐S. Zhang , S.‐Y. Chen , Plant J. 2007, 52, 716. 17877700 10.1111/j.1365-313X.2007.03268.x

[60] C. Gatz , Mol. Plant. Microbe. Interact. 2013, 26, 151. 23013435 10.1094/MPMI-04-12-0078-IA

[61] Y. Zhang , W. Fan , M. Kinkema , X. Li , X. Dong , Proc. Natl. Acad. Sci. USA 1999, 96, 6523. 10339621 10.1073/pnas.96.11.6523 PMC26915

[62] Y. Zhang , M. J. Tessaro , M. Lassner , X. Li , Plant Cell 2003, 15, 2647. 14576289 10.1105/tpc.014894 PMC280568

[63] P. Qi , M. Huang , X. Hu , Y. Zhang , Y. Wang , P. Li , S. Chen , D. Zhang , S. Cao , W. Zhu , J. Xie , J. Cheng , Y. Fu , D. Jiang , X. Yu , B. Li , Plant Cell. 2022, 34, 1666. 35043960 10.1093/plcell/koac015 PMC9048914

[64] H. W. Kim , M. Wang , J. Nat. Prod. 2021, 84, 2795. 34662515 10.1021/acs.jnatprod.1c00399 PMC8631337

[65] M. C. Schruff , M. Spielman , S. Tiwari , S. Adams , N. Fenby , R. J. Scott , Development 2006, 133, 251. 16339187 10.1242/dev.02194

[66] G. Hagen , T. Guilfoyle , Plant Mol. Biol. 2002, 49, 373. 12036261

[67] Y. Okushima , I. Mitina , H. L. Quach , A. Theologis , Plant J. 2005, 43, 29. 15960614 10.1111/j.1365-313X.2005.02426.x

[68] S. Pien , U. Grossniklaus , Biochim. Biophys. Acta 2007, 1769, 375. 17363079 10.1016/j.bbaexp.2007.01.010

[69] E. Sornay , C. Forzani , M. Forero‐Vargas , W. Dewitte , J. A. Murray , Plant J. 2015, 84, 41. 26261067 10.1111/tpj.12957 PMC5102630

[70] M. Ingouff , P. E. Jullien , F. Berger , Plant Cell 2006, 18, 3491. 17172356 10.1105/tpc.106.047266 PMC1785409

[71] L. Du , N. Li , L. Chen , Y. Xu , Y. Li , Y. Zhang , C. Li , Y. Li , Plant Cell 2014, 26, 665. 24585836 10.1105/tpc.114.122663 PMC3967032

[72] I. Rauluseviciute , R. Riudavets‐Puig , R. Blanc‐Mathieu , J. A. Castro‐Mondragon , K. Ferenc , V. Kumar , R. B. Lemma , J. Lucas , J. Chèneby , D. Baranasic , A. Khan , Nucleic Acids Res. 2024, 52, D174. 37962376 10.1093/nar/gkad1059 PMC10767809

[73] K. Yano , E. Yamamoto , K. Aya , H. Takeuchi , P.‐C. Lo , L. Hu , M. Yamasaki , S. Yoshida , H. Kitano , K. Hirano , M. Matsuoka , Nat. Genet. 2016, 48, 927. 27322545 10.1038/ng.3596

[74] X. Huang , Y. Zhao , X. Wei , C. Li , A. Wang , Q. Zhao , W. Li , Y. Guo , L. Deng , C. Zhu , D. Fan , Y. Lu , Q. Weng , K. Liu , T. Zhou , Y. Jing , L. Si , G. Dong , T. Huang , T. Lu , Q. Feng , Q. Qian , J. Li , B. Han , Nat. Genet. 2012, 44, 32. 10.1038/ng.1018 22138690

[75] X. Huang , X. Wei , T. Sang , Q. Zhao , Q. Feng , Y. Zhao , C. Li , C. Zhu , T. Lu , Z. Zhang , M. Li , D. Fan , Y. Guo , A. Wang , L. Wang , L. Deng , W. Li , Y. Lu , Q. Weng , K. Liu , T. Huang , T. Zhou , Y. Jing , W. Li , Z. Lin , E. S. Buckler , Q. Qian , Q.‐F. Zhang , J. Li , B. Han , Nat. Genet. 2010, 42, 961. 20972439 10.1038/ng.695

[76] J. Lisec , R. C. Meyer , M. Steinfath , H. Redestig , M. Becher , H. Witucka‐Wall , O. Fiehn , O. Törjék , J. Selbig , T. Altmann , L. Willmitzer , Plant J. 2008, 53, 960. 18047556 10.1111/j.1365-313X.2007.03383.x PMC2268983

[77] N. Li , J. Shi , X. Wang , G. Liu , H. Wang , BMC Plant Biol. 2014, 14, 114. 24779415 10.1186/1471-2229-14-114 PMC4021082

[78] H. Wang , M. Yan , M. Xiong , P. Wang , Y. Liu , Q. Xin , L. Wan , G. Yang , D. Hong , Theor. Appl. Genet. 2020, 133, 1321. 32002584 10.1007/s00122-020-03553-9

[79] Y. Yang , K. Zhu , H. Li , S. Han , Q. Meng , S. U. Khan , C. Fan , K. Xie , Y. Zhou , Plant Biotechnol. J. 2018, 16, 1322. 29250878 10.1111/pbi.12872 PMC5999189

[80] Y. G. Xiao , Q. B. Sun , X. J. Kang , C. B. Chen , M. Ni , New Phytol. 2016, 209, 636. 26389843 10.1111/nph.13632

[81] P. A. Quijada , J. A. Udall , B. Lambert , T. C. Osborn , Theor. Appl. Genet. 2006, 113, 549. 16767447 10.1007/s00122-006-0323-1

[82] G. Cai , Q. Yang , Q. Yang , Z. Zhao , H. Chen , J. Wu , C. Fan , Y. Zhou , BMC Genet. 2012, 13, 105. 23216693 10.1186/1471-2156-13-105 PMC3575274

[83] H. Dong , C. Tan , Y. Li , Y. He , S. Wei , Y. Cui , Y. Chen , D. Wei , Y. Fu , Y. He , H. Wan , Z. Liu , Q. Xiong , K. Lu , J. Li , W. Qian , Front. Plant. Sci. 2018, 9, 921. 30073005 10.3389/fpls.2018.00921 PMC6058094

[84] K. Lu , L. Peng , C. Zhang , J. Lu , B. Yang , Z. Xiao , Y. Liang , X. Xu , C. Qu , K. Zhang , L. Liu , Q. Zhu , M. Fu , X. Yuan , J. Li , Front. Plant. Sci. 2017, 8, 206. 28261256 10.3389/fpls.2017.00206 PMC5309214

[85] I. Blaženović , T. Kind , J. Ji , O. Fiehn , Metabolites 2018, 8, 31. 29748461 10.3390/metabo8020031 PMC6027441

[86] T. Kind , H. Tsugawa , T. Cajka , Y. Ma , Z. Lai , S. S. Mehta , G. Wohlgemuth , D. K. Barupal , M. R. Showalter , M. Arita , O. Fiehn , Mass. Spectrom. Rev. 2018, 37, 513. 28436590 10.1002/mas.21535 PMC8106966

[87] R. Chaleckis , I. Meister , P. Zhang , C. E. Wheelock , Curr. Opin. Biotechnol. 2019, 55, 44. 30138778 10.1016/j.copbio.2018.07.010

[88] B. Chalhoub , F. Denoeud , S. Liu , I. A. P. Parkin , H. Tang , X. Wang , J. Chiquet , H. Belcram , C. Tong , B. Samans , M. Corréa , C. Da Silva , J. Just , C. Falentin , C. S. Koh , I. Le Clainche , M. Bernard , P. Bento , B. Noel , K. Labadie , A. Alberti , M. Charles , D. Arnaud , H. Guo , C. Daviaud , S. Alamery , K. Jabbari , M. Zhao , P. P. Edger , H. Chelaifa , et al., Science 2014, 345, 950. 25146293 10.1126/science.1253435

[89] S. Purcell , B. Neale , K. Todd‐Brown , L. Thomas , M. A. R. Ferreira , D. Bender , J. Maller , P. Sklar , P. I. W. de Bakker , M. J. Daly , P. C. Sham , Am. J. Hum. Genet. 2007, 81, 559. 17701901 10.1086/519795 PMC1950838

[90] C. Widmer , C. Lippert , O. Weissbrod , N. Fusi , C. Kadie , R. Davidson , J. Listgarten , D. Heckerman , Sci. Rep. 2014, 4, 6874. 25387525 10.1038/srep06874 PMC4230738

[91] A. L. Harper , M. Trick , J. Higgins , F. Fraser , L. Clissold , R. Wells , C. Hattori , P. Werner , I. Bancroft , Nat. Biotechnol. 2012, 30, 798. 22820317 10.1038/nbt.2302

[92] P. Langfelder , S. Horvath , BMC Bioinformatics 2008, 9, 559. 19114008 10.1186/1471-2105-9-559 PMC2631488

[93] X. Gao , P. Yan , W. Shen , X. Li , P. Zhou , Y. Li , Anal. Biochem. 2013, 437, 172. 23499974 10.1016/j.ab.2013.02.028

[94] C. Dai , Y. Li , L. Li , Z. Du , S. Lin , X. Tian , S. Li , B. Yang , W. Yao , J. Wang , L. Guo , S. Lu , Mol. Breeding 2020, 40, 96.

[95] S. D. Yoo , Y. H. Cho , J. Sheen , Nat. Protoc. 2007, 2, 1565. 17585298 10.1038/nprot.2007.199

[96] P. Ewels , M. Magnusson , S. Lundin , M. Käller , Bioinformatics 2016, 32, 3047. 27312411 10.1093/bioinformatics/btw354 PMC5039924

[97] A. Dobin , C. A. Davis , F. Schlesinger , J. Drenkow , C. Zaleski , S. Jha , P. Batut , M. Chaisson , T. R. Gingeras , Bioinformatics 2013, 29, 15. 23104886 10.1093/bioinformatics/bts635 PMC3530905

[98] B. Li , C. N. Dewey , BMC Bioinformatics 2011, 12, 323. 21816040 10.1186/1471-2105-12-323 PMC3163565

[99] M. I. Love , W. Huber , S. Anders , Genome Biol. 2014, 15, 550. 25516281 10.1186/s13059-014-0550-8 PMC4302049

[100] X. Han , X. Wu , Plant Cell 2025, 37.

[101] H. Li , R. Durbin , Bioinformatics 2010, 26, 589. 20080505 10.1093/bioinformatics/btp698 PMC2828108

[102] Y. Zhang , T. Liu , C. A. Meyer , J. Eeckhoute , D. S. Johnson , B. E. Bernstein , C. Nusbaum , R. M. Myers , M. Brown , W. Li , X. S. Liu , Genome Biol. 2008, 9, R137. 18798982 10.1186/gb-2008-9-9-r137 PMC2592715

[103] F. Ramírez , V. Bhardwaj , Nat. Commun. 2018, 9, 189. 29335486 10.1038/s41467-017-02525-w PMC5768762

[104] T. L. Bailey , J. Johnson , C. E. Grant , W. S. Noble , Nucleic Acids Res. 2015, 43, W39. 25953851 10.1093/nar/gkv416 PMC4489269

