# Supplementary material for: Marker Metabolite‐Based Multi‐Omics Analysis Identifies New Loci Controlling Thousand Seed Weight in Brassica Napus
Source: Adv Sci (Weinh). 2025 Sep 19;12(42):e12509. doi: 10.1002/advs.202512509 (PMC12622543; doi:10.1002/advs.202512509)
Supplement: Supplementary file 1 — Supporting Information [file ADVS-12-e12509-s001.pdf]

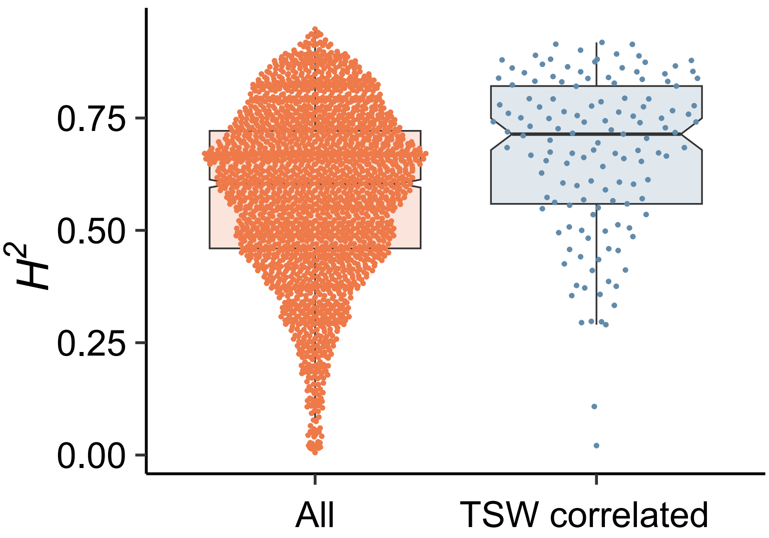

Supplementary Fig 1 Distribution of the values of broad-sense heritability (H^2) of 2,172 metabolites and 137 TSW-correlated metabolites (p = 2.2×10^{-16}).

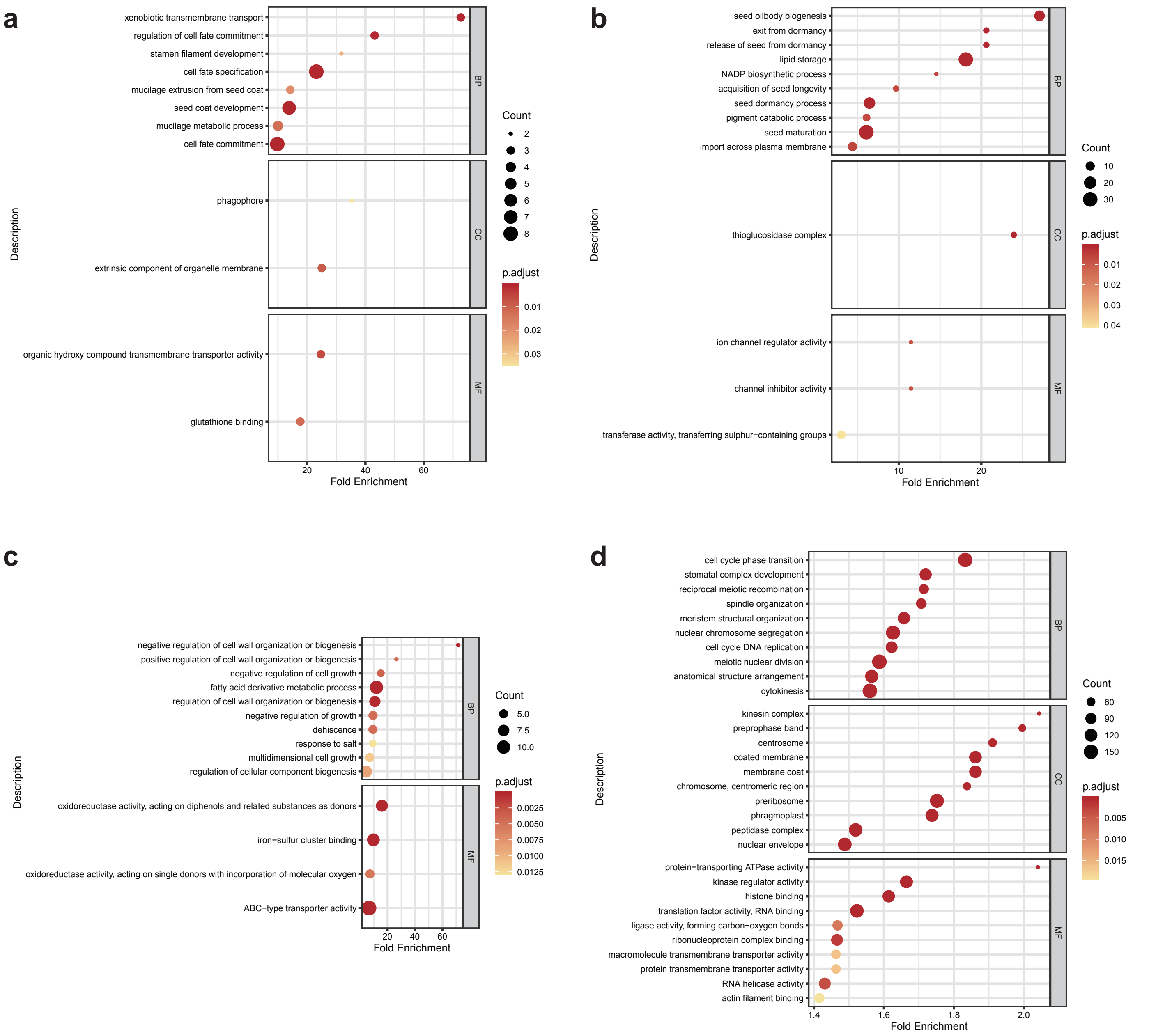

Supplementary Fig 2 Enrichment analysis for genes in modules significantly correlated with TSW. **a** Enrichment analysis for genes in Module sienna3. **b** Enrichment analysis for genes in Module blue. **c** Enrichment analysis for genes in Module cyan. **d** Enrichment analysis for genes in Module turquoise.

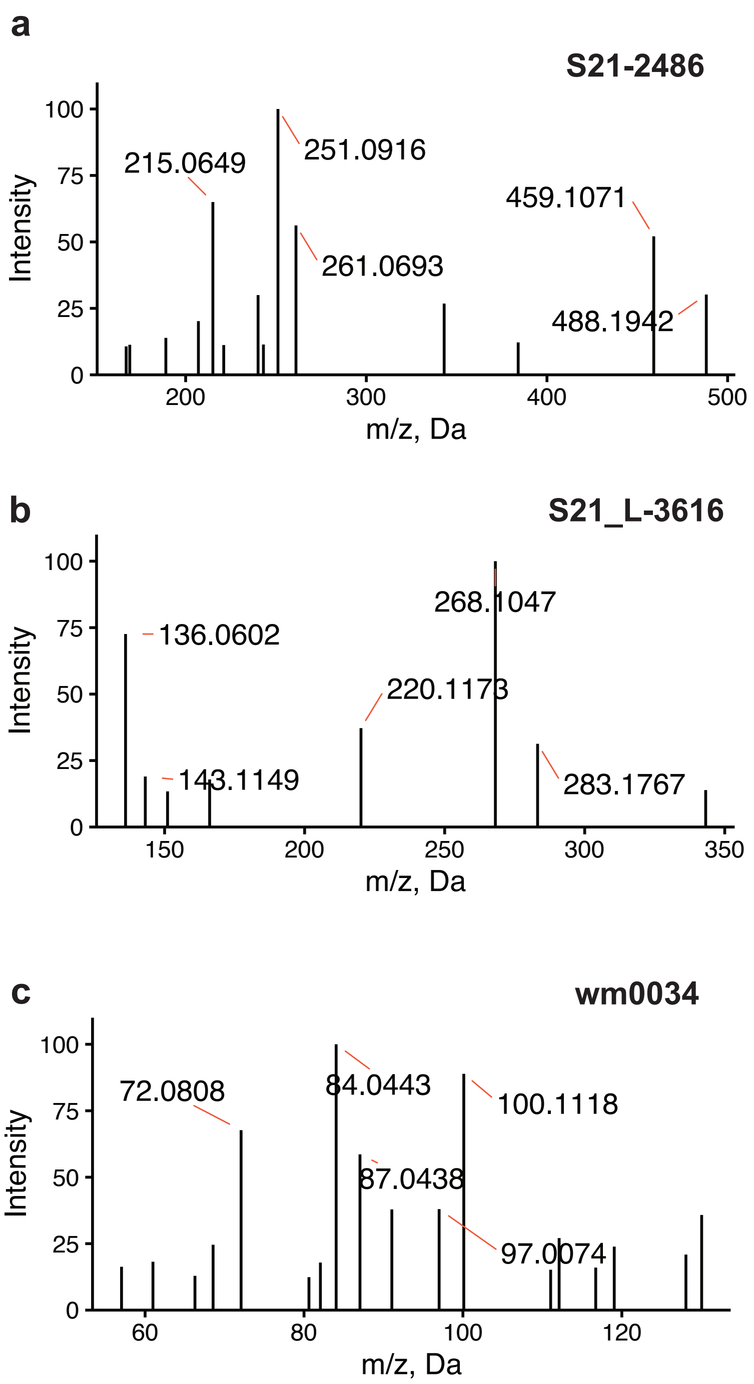

Supplementary Fig 3 *mQTL4855* metabolites high-resolution mass spectrum plots[12]. **a** S21-2486. **b** S21_L-3616. **c** wm0034.

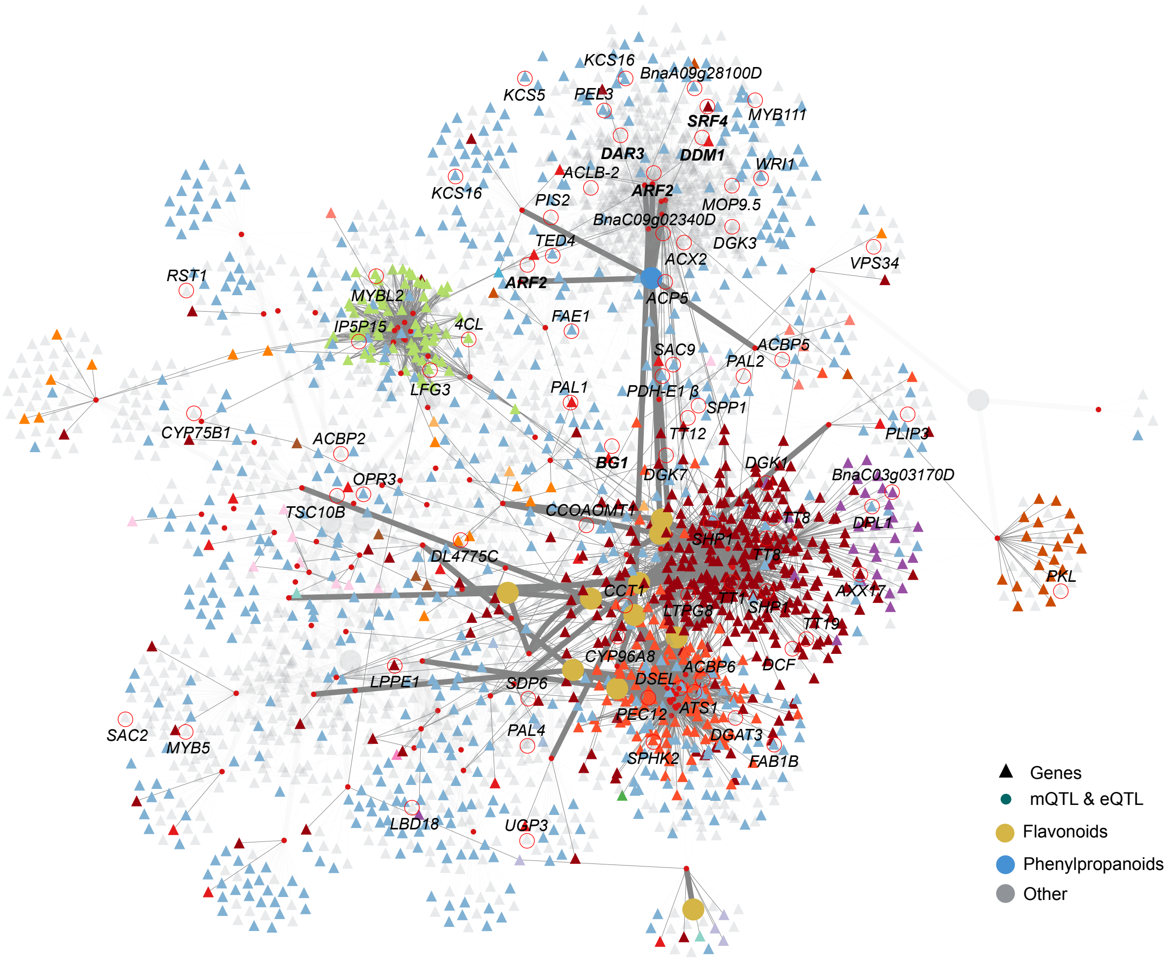

Supplementary Fig 4 Network built based on the correlation among seed oil content and TSW-correlated metabolites, genes, and QTLs. Genes are shown as triangles with a distinct color per co-expression module. All modules significantly correlated with TSW are shown in this network. The QTLs are detected simultaneously by the significant associated genes (eQTL) and metabolites (mQTL) in this triple relationship network.

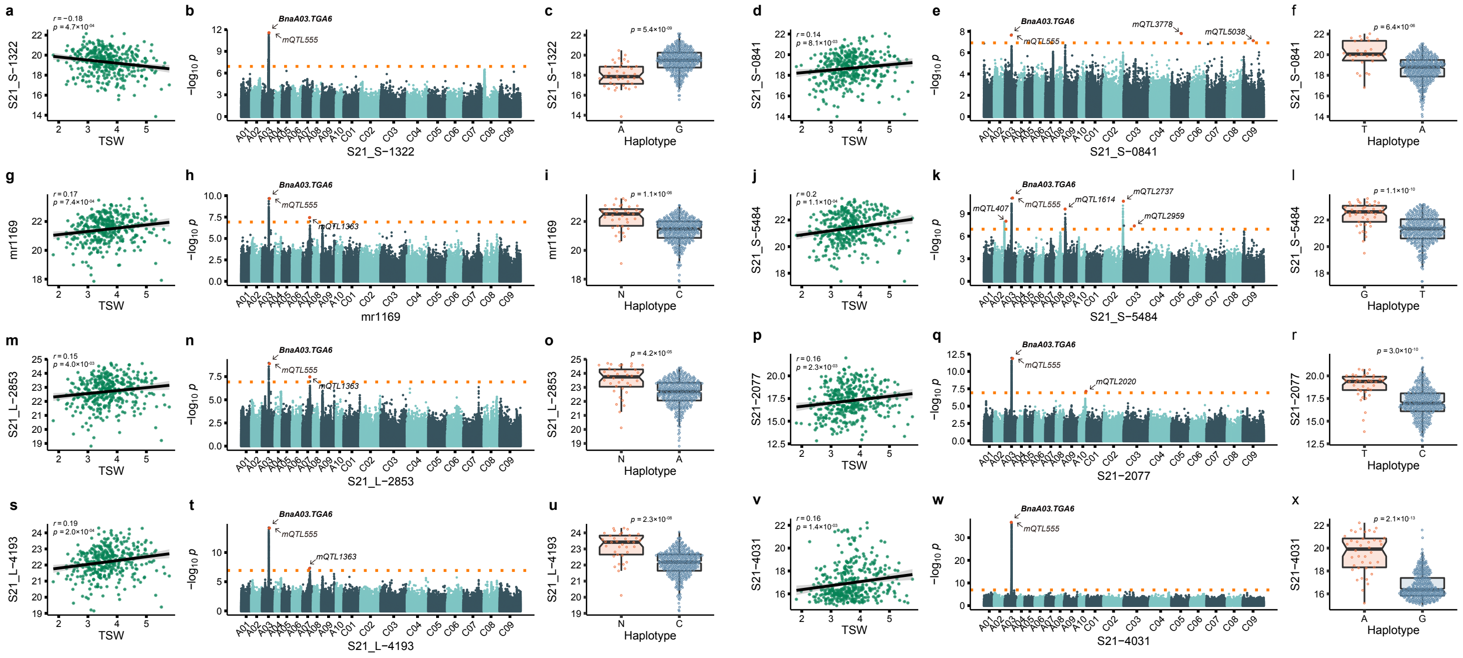

Supplementary Fig 5 mGWAS results related to the loci on chromosome A03 (position = 15,521,736 bp) (2017). Correlation analysis between TSW and **a** S21_S-1322, **d** S21_S-0841, **g** mr1169, **j** S21_S-5484, **m** S21_L−2853, **p** S21-2077 **s** S21_L−4193 **v** S21-4031. Manhattan plot of mGWAS for **b** S21_S-1322, **e** S21_S-0841, **h** mr1169, **k** S21_S-5484, **n** S21_L−2853, **q** S21-2077 **t** S21_L−4193 **w** S21-4031. Haplotype analysis of the lead variation in *mQTL555* for **c** S21_S-1322, **f** S21_S-0841, **i** mr1169, **l** S21_S-5484, **o** S21_L−2853, **r** S21-2077 **u** S21_L−4193 **x** S21-4031.

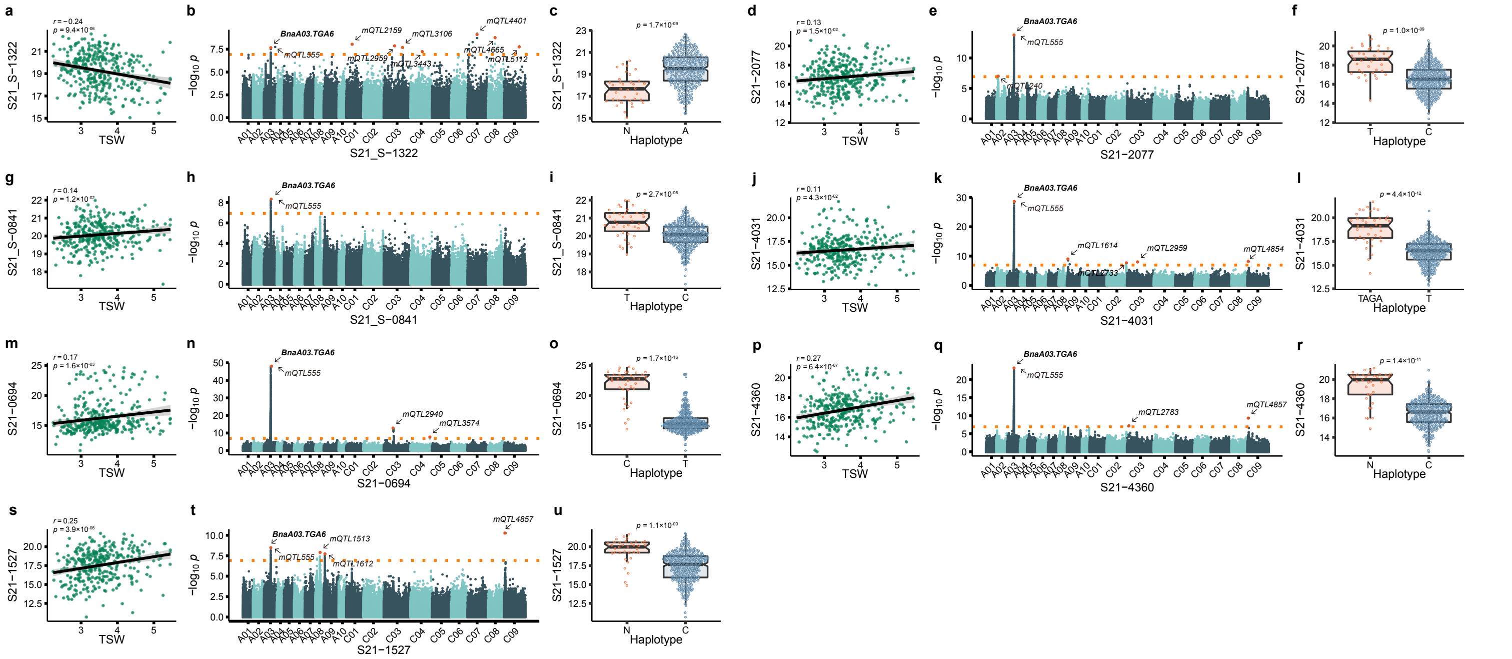

Supplementary Fig 6 mGWAS results related to the loci on chromosome A03 (position = 15,521,736 bp) (2018). Correlation analysis between TSW and **a** S21_S-1322, **d** S21-2077, **g** S21_S-0841, **j** S21-4031, **m** S21-0694, **p** S21-4360, **s** S21-1527. Manhattan plot of mGWAS for **b** S21_S-1322, **e** S21-2077, **h** S21_S-0841, **k** S21-4031, **n** S21-0694, **q** S21-4360, **t** S21-1527. Haplotype analysis of the lead variation in *mQTL555* for **c** S21_S-1322, **f** S21-2077, **i** S21_S-0841, **l** S21-4031, **o** S21-0694, **r** S21-4360, **u** S21-1527.

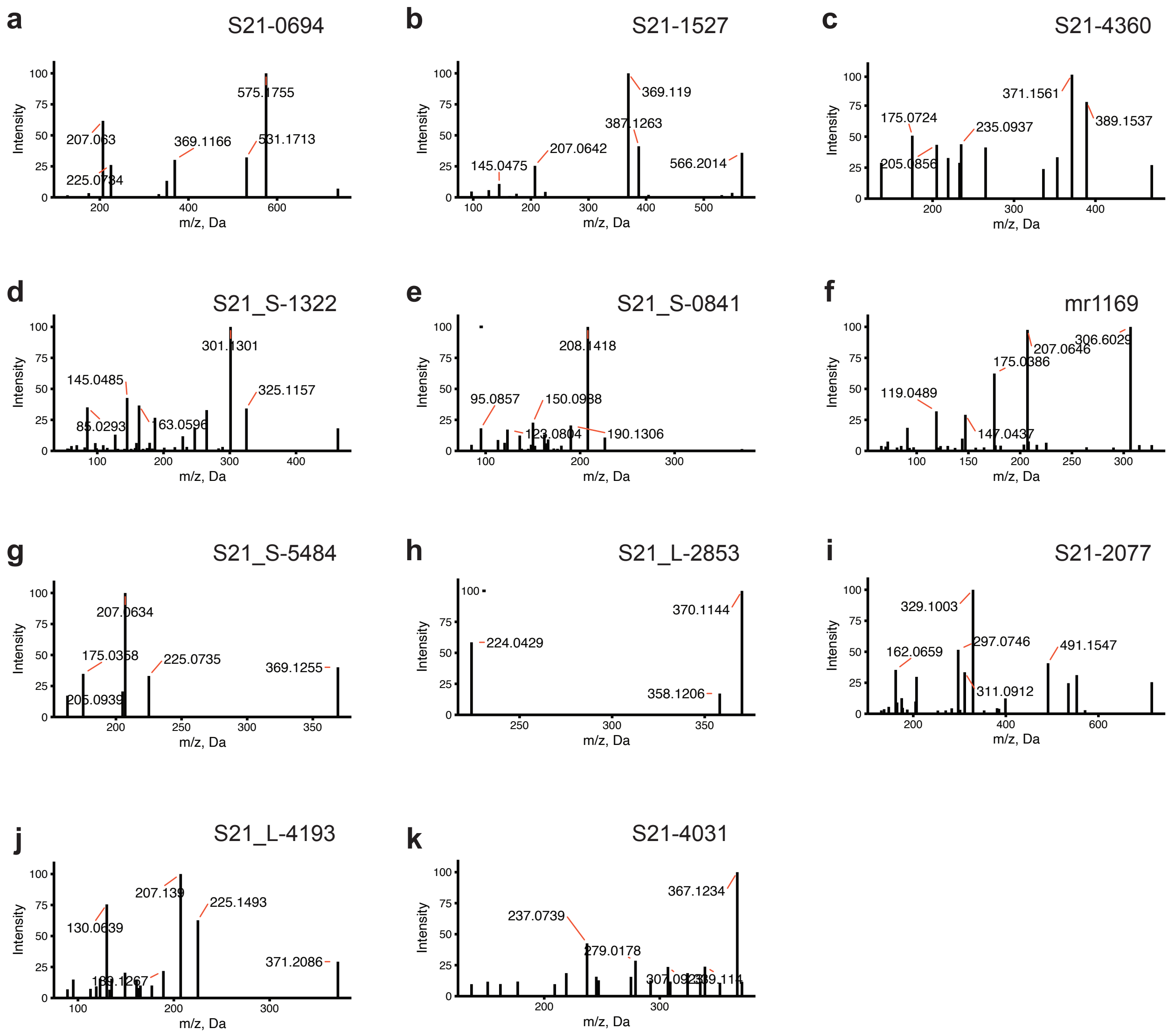

Supplementary Fig 7 *mQTL555* metabolites high-resolution mass spectrum plots[12]. **a** S21-0694. **b** S21-1527. **c** S21-4360. **d** S21_S-1322. **e** S21_S-0841. **f** mr1169. **g** S21_S-5484. **h** S21_L−2853. **i** S21-2077. j S21_L−4193. **k** S21-4031.

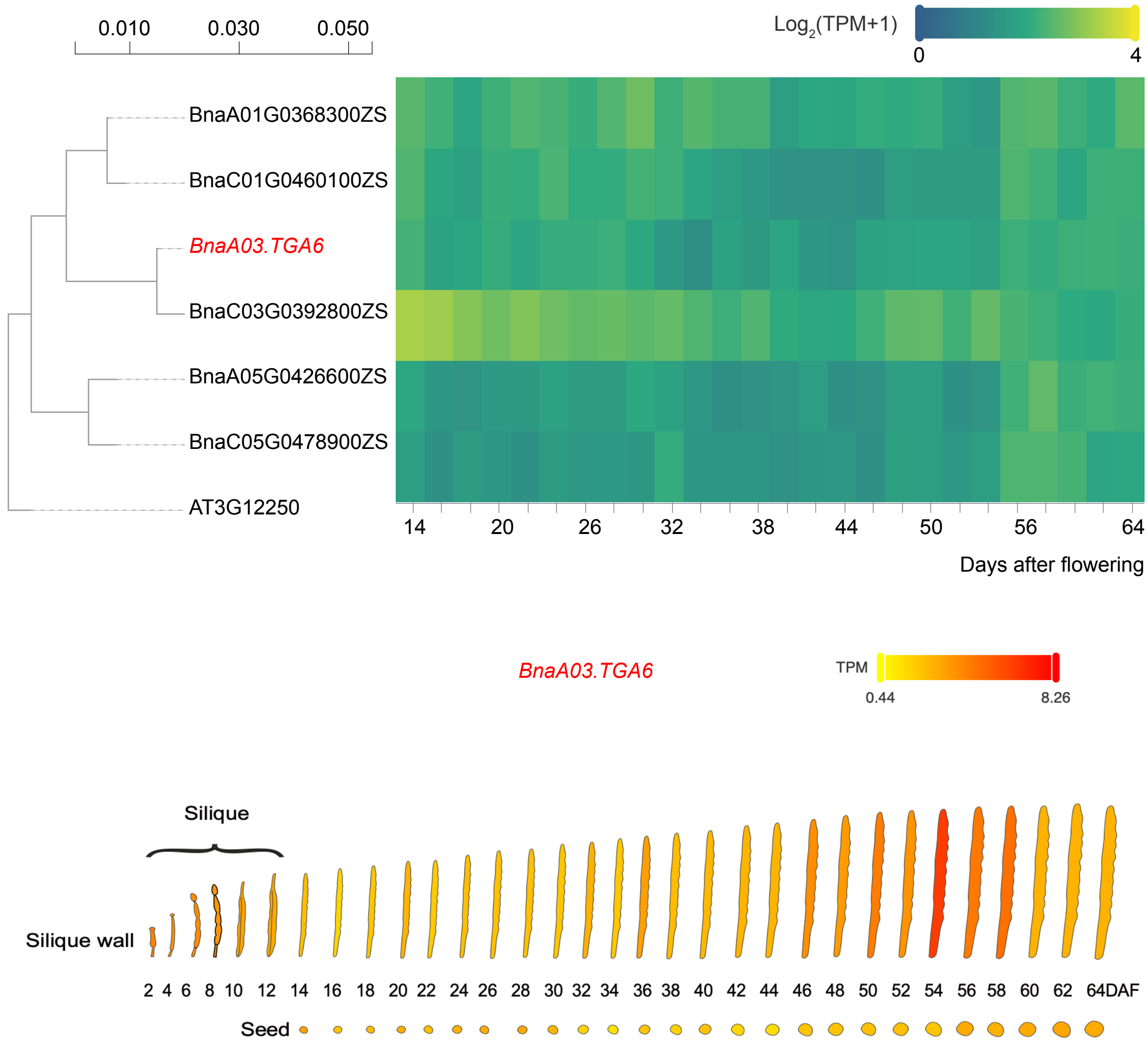

Supplementary Fig 8 *BnaA03.TGA6* genes expression pattern in developing seeds and siliques, and its homolog genes expression pattern in developing seeds. Expression data is from BnTIR (https://yanglab.hzau.edu.cn/BnIR)[89].

a

Target	Chr	Line		Sequence	PAM	type
Target 1	A03	L9	T	AGGCTTGCTCAAAATCGAG	AGG	none
Target 1	A03	L22	T	AGGCTTGCTCAAAATCGAG	AGG	none
Target 1	A03	WT	T	AGGCTTGCTCAAAATCGAG	AGG	
Target 2	A03	L9	A	TAGGCGTATGTT---CAGC	TGG	deletion
Target 2	A03	L22	A	TAGGCGTATGTTCAGCAAGC	TGG	insertion
Target 2	A03	WT	A	TAGGCGTATGTTCAGCAGC	TGG	

b

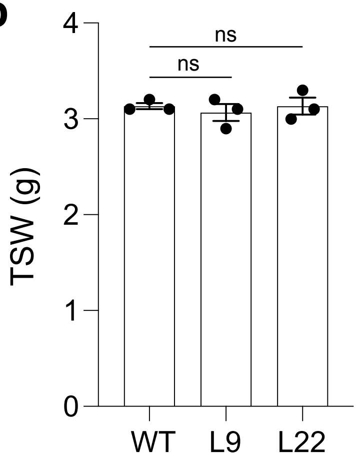

Supplementary Fig 9 TSW phenotype of the single *BnaA03.TGA6* knockout mutant seeds. **a** *BnaA03.TGA6* single mutants genotypes. **b** *BnaA03.TGA6* single mutants TSW. Values are means ± s.e.m., n = 3. Statistical analysis is using Student's t-test (ns, not significant).

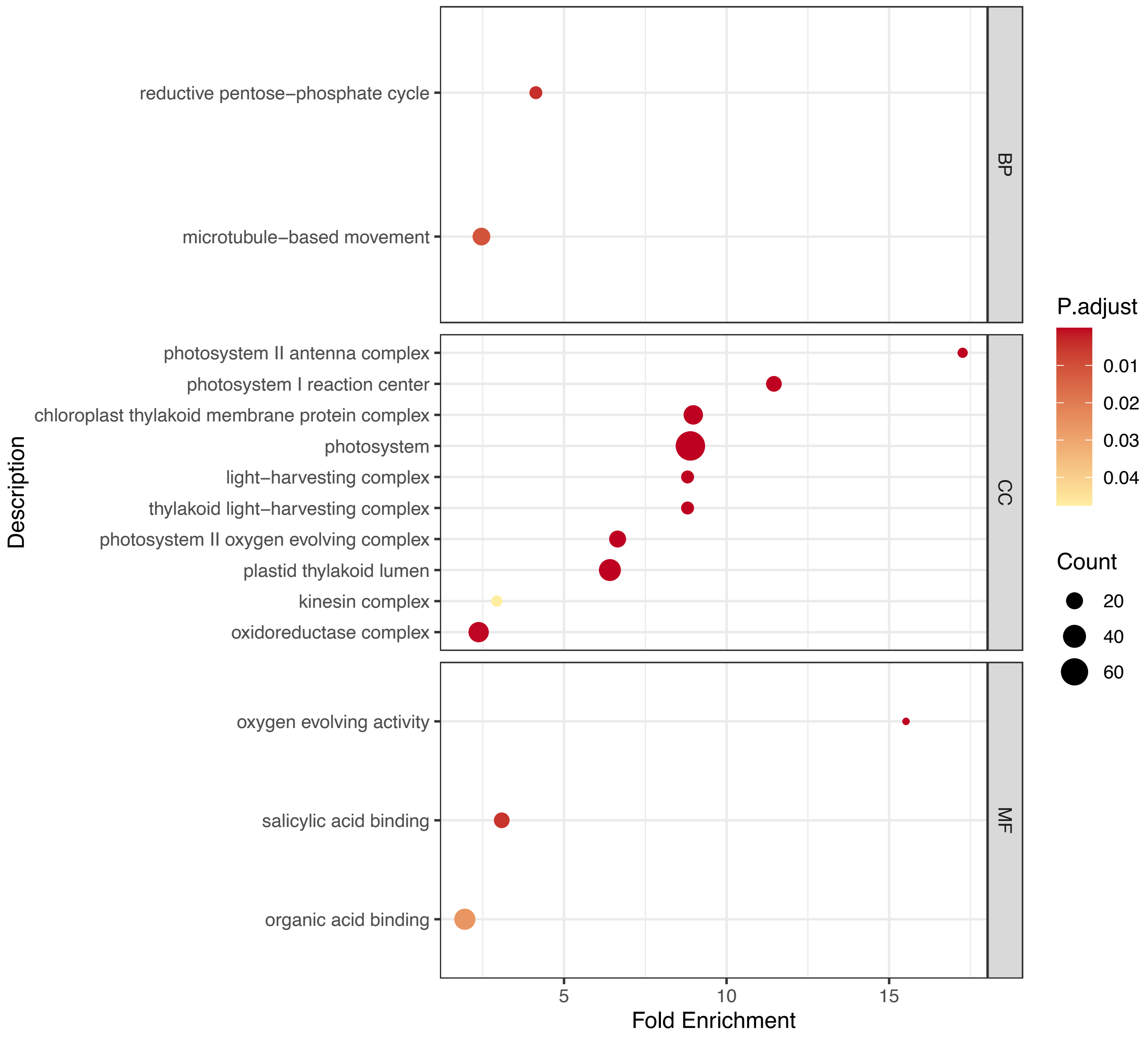

Supplementary Figure 10 Enrichment analysis for genes that are significantly different between WT and *tga6.*

S21-1527

a

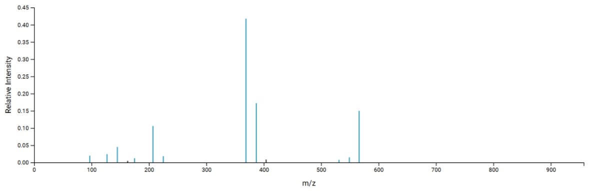

b

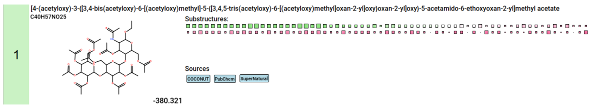

S21-4360

c

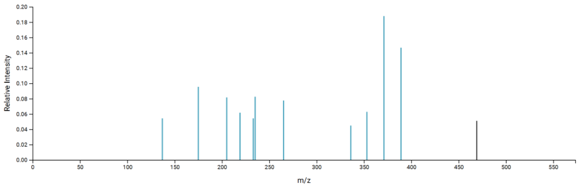

d

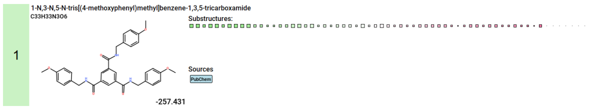

S21-0694

e

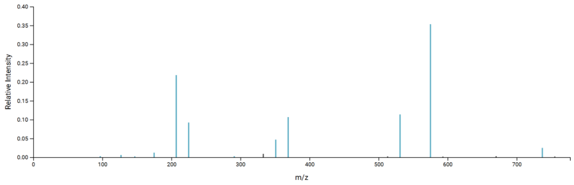

f

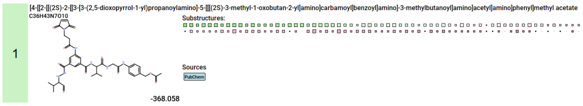

Supplementary Figure 11 Predicted structures for three metabolites that are significantly increased in *tga6* mutants seeds. **a** Relative intensity for S21-1527. **b** Predicted structure for S21-1527. **c** Relative intensity for S21-4360. **d** Predicted structure for S21-4360. **e** Relative intensity for S21-0694. **f** Predicted structure for S21-0694.

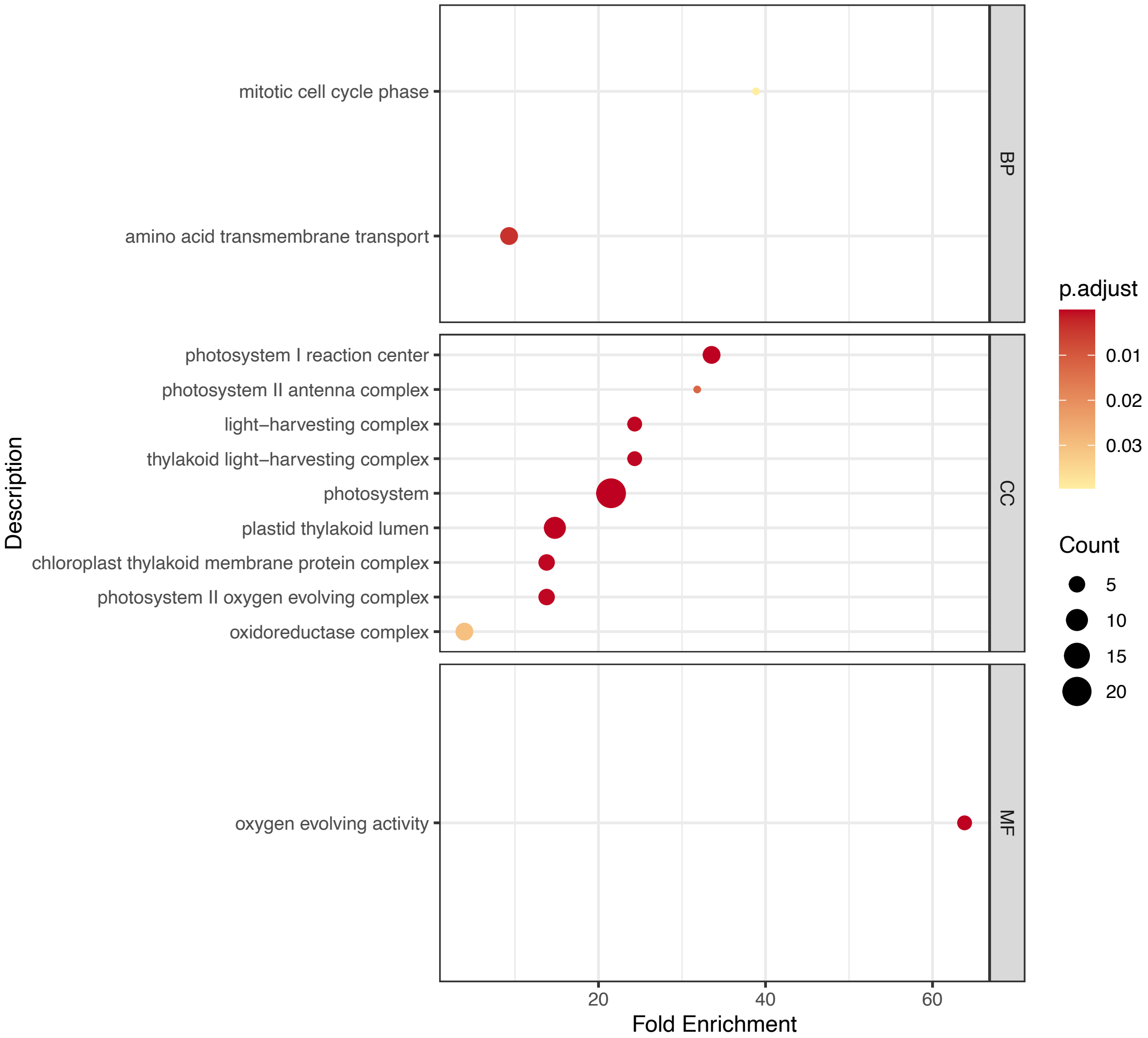

Supplementary Figure 12 Enrichment analysis for genes that are *tga6* 40DAF transcriptome DEGs and BnaA03.TGA6 CUT&Tag hits within promoter region 2kb.

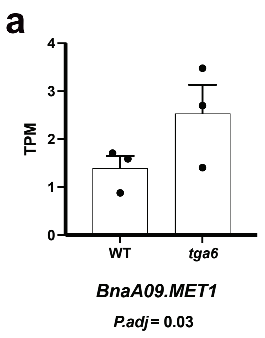

b

Gene_ID	Gene_name	*cis* Number
BnaA06g13370D	*DA1*	0
BnaC05g14930D	*DA1*	0
BnaA08g22120D	*DA1*	5
BnaC08g18690D	*DA1*	5
BnaA09g42250D	*MET1*	1
BnaA01g13440D	*TTG2*	1
BnaC01g18920D	*TOP1-α*	4

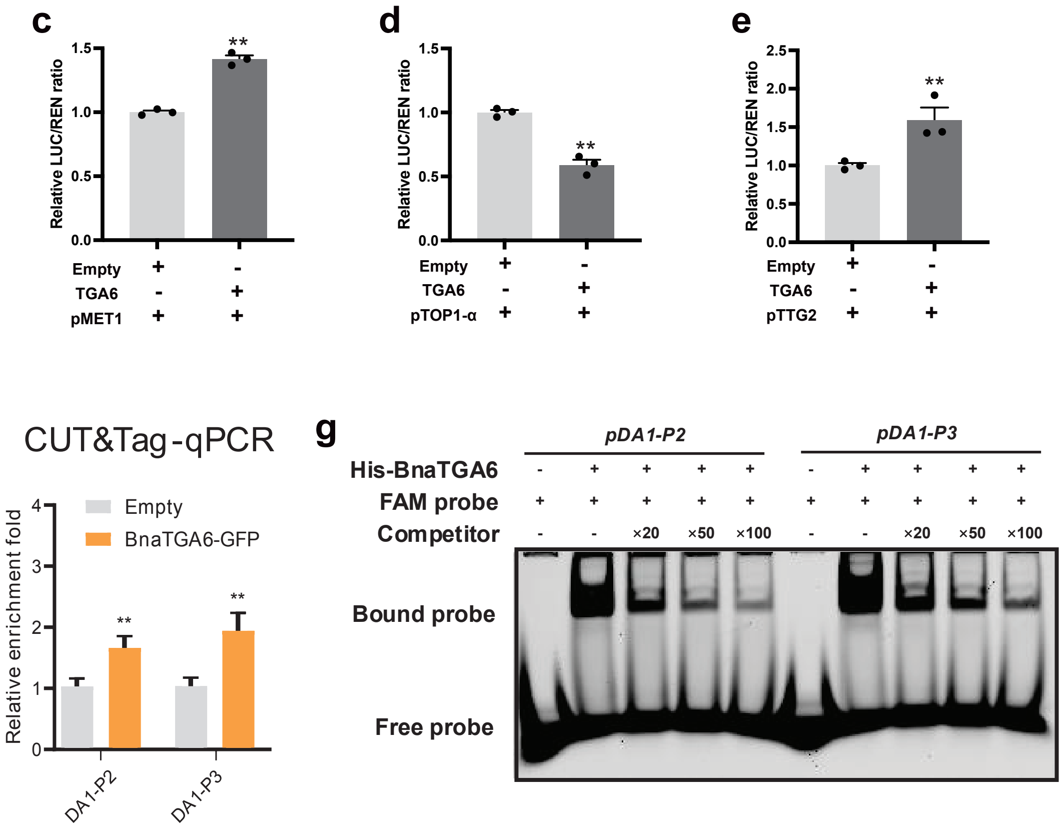

Supplementary Figure 13 BnaA03.TGA6 downstream targets validation. **a** Expression level of *BnaA09.MET1* in WT and *tga6* mutant developing seed (40DAF). **b** Predicted BnaA03.TAG6 binding motifs. **c** Bar graph showing the relative *LUC/REN* ratio from the dual-luciferase assay, indicating the transcriptional activation of *BnaA09.MET1* by BnaA03.TGA6, **d** *BnaC01.TOP1-α,* **e** *BnaA01.TTG2.* **f** CUT&Tag-qPCR results showing *in vivo* binding of BnaA03.TGA6 to the *BnaA08.DA1* promoter region. **g** EMSA results demonstrating that the His-BnaA03.TGA6 fusion protein directly binds to specific motifs within the *BnaA08.DA1* promoter.
